# SVM-RFE: selection and visualization of the most relevant features through non-linear kernels

**DOI:** 10.1186/s12859-018-2451-4

**Published:** 2018-11-19

**Authors:** Hector Sanz, Clarissa Valim, Esteban Vegas, Josep M. Oller, Ferran Reverter

**Affiliations:** 1 Department of Genetics, Microbiology and Statistics, Faculty of Biology, Universitat de Barcelona, Diagonal, 643, 08028 Barcelona, Catalonia Spain; 2 Department of Osteopathic Medical Specialties, Michigan State University, 909 Fee Road, Room B 309 West Fee Hall, East Lansing, MI 48824 USA; 3 Department of Immunology and Infectious Diseases, Harvard T.H. Chen School of Public Health, 675 Huntington Ave, Boston, MA 02115 USA; 4 Centre for Genomic Regulation (CRG), The Barcelona Institute for Science and Technology, Dr. Aiguader 88, 08003 Barcelona, Spain

**Keywords:** Support vector machines, Relevant variables, Recursive feature elimination, Kernel methods

## Abstract

**Background:**

Support vector machines (SVM) are a powerful tool to analyze data with a number of predictors approximately equal or larger than the number of observations. However, originally, application of SVM to analyze biomedical data was limited because SVM was not designed to evaluate importance of predictor variables. Creating predictor models based on only the most relevant variables is essential in biomedical research. Currently, substantial work has been done to allow assessment of variable importance in SVM models but this work has focused on SVM implemented with linear kernels. The power of SVM as a prediction model is associated with the flexibility generated by use of non-linear kernels. Moreover, SVM has been extended to model survival outcomes. This paper extends the Recursive Feature Elimination (RFE) algorithm by proposing three approaches to rank variables based on non-linear SVM and SVM for survival analysis.

**Results:**

The proposed algorithms allows visualization of each one the RFE iterations, and hence, identification of the most relevant predictors of the response variable. Using simulation studies based on time-to-event outcomes and three real datasets, we evaluate the three methods, based on pseudo-samples and kernel principal component analysis, and compare them with the original SVM-RFE algorithm for non-linear kernels. The three algorithms we proposed performed generally better than the gold standard RFE for non-linear kernels, when comparing the truly most relevant variables with the variable ranks produced by each algorithm in simulation studies. Generally, the RFE-pseudo-samples outperformed the other three methods, even when variables were assumed to be correlated in all tested scenarios.

**Conclusions:**

The proposed approaches can be implemented with accuracy to select variables and assess direction and strength of associations in analysis of biomedical data using SVM for categorical or time-to-event responses. Conducting variable selection and interpreting direction and strength of associations between predictors and outcomes with the proposed approaches, particularly with the RFE-pseudo-samples approach can be implemented with accuracy when analyzing biomedical data. These approaches, perform better than the classical RFE of Guyon for realistic scenarios about the structure of biomedical data.

**Electronic supplementary material:**

The online version of this article (10.1186/s12859-018-2451-4) contains supplementary material, which is available to authorized users.

## Background

Analysis of investigations aiming to classify or predict response variables in biomedical research oftentimes is challenging because of data sparsity generated by limited sample sizes and a moderate or very large number of predictors. Moreover, in biomedical research, it is particularly relevant to learn about the relative importance of predictors to shed light in mechanisms of association or to save costs when developing biomarkers and surrogates. Each marker included in an assay increases the price of the biomarker and several technologies used to measure biomarkers can accommodate a limited number of markers. Support Vector Machine (SVM) models are a powerful tool to identify predictive models or classifiers, not only because they accommodate well sparse data but also because they can classify groups or create predictive rules for data that cannot be classified by linear decision functions. In spite of that, SVM has only recently became popular in the biomedical literature, partially because SVMs are complex and partially because SVMs were originally geared towards creating classifiers based on all available variables, and did not allow assessing variable importance.

Currently, there are three categories of methods to assess importance of variables in SVM: filter, wrapper, and embedded methods. The problem with the existing approaches within these three categories is that they are mainly based on SVM with linear kernels. Therefore, the existing methods do not allow implementing SVM in data that cannot be classified by linear decision functions. The best approaches to work with non-linear kernels are wrapper methods because filter methods are less efficient than wrapper methods and embedded methods are focused on linear kernels. The gold standard of wrapper methods is recursive feature elimination (RFE) proposed by Guyon et al. [1]. Although wrapper methods outweigh other procedures, there is no approach implemented to visualize RFE results. The RFE algorithm for non-linear kernels allows ranking variables but not comparing the performance of all variables in a specific iteration, i.e., interpreting results in terms of: association with the response variable, association with the other variables and magnitude of this association, which is a key point in biomedical research. Moreover, previous work with the RFE algorithm for non-linear kernels has generally focused on classification and disregarded time-to-event responses with censoring that are common in biomedical research.

The work presented in this article expands RFE to visualize variable importance in the context of SVM with non-linear kernels and SVM for survival responses. More specifically, we propose: i) a RFE-based algorithm that allows visualization of variable importance by plotting the predictions of the SVM model; and ii) two variants from the RFE-algorithms based on representation of variables into a multidimensional space such as the KPCA space. In the first section, we briefly review existing methods to evaluate importance of variables by ranking, by selecting variables, and by allowing visualization of variable relative importance. In the Methods section, we present our proposed approaches and extensions. Next, in Results, we evaluate the proposed approaches using simulated data and three real datasets. Finally, we discuss the main characteristics and obtained results of all three proposed methods.

### Existing approaches to assess variable importance

The approaches to assess variable importance in SVM can be grouped in filter, embedded and wrapper method classes. Filter methods assess the relevance of variables by looking only at the intrinsic properties of the data without taking into account any information provided by the classification algorithm. In other words, they perform variable selection before fitting the learning algorithm. In most cases, a variable relevance score is calculated, and low-scoring variables are removed. Afterwards, the “relevant” variable subset is input into the classification algorithm. Filter methods include the F-score [2, 3].

Embedded methods, are built into a classifier and, thus, are specific to a given learning algorithm. In the SVM framework, all embedded methods are limited to linear kernels. Additionally, most of these methods are based on a somewhat penalization term, i.e., variables are penalized depending on their values with some methods explicitly constraining the number of variables, and others penalizing the number of variables [4, 5]. An additional exact algorithm was developed for SVM in classification problems using the Benders decomposition algorithm [6]. Finally, a penalized version of the SVM with different penalization terms was suggested by Becker et al. [7, 8]

**Fig. 1:**
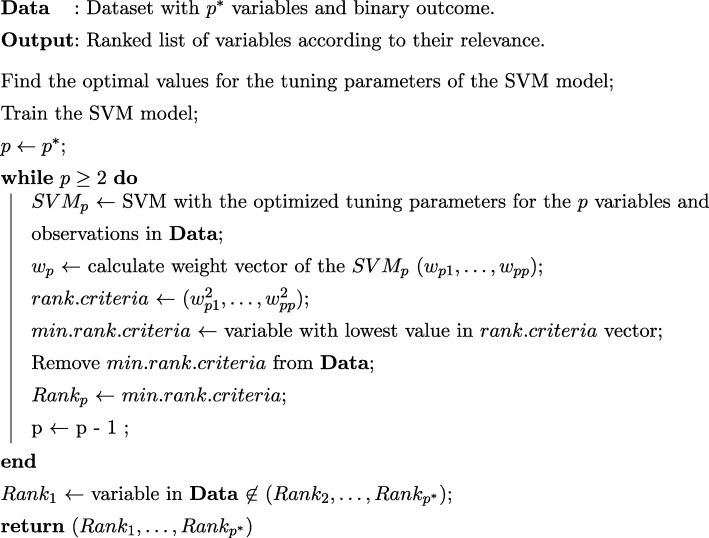
Pseudo-code of the SVM-RFE algorithm using the linear kernel in a model for binary classification

Wrapper methods evaluate a specific subset of variables by training and testing a specific classification model, and are thus, tailored to a specific classification algorithm. The idea is to search the space of all variable subsets with an algorithm wrapped around the classification model. However, as the space of variables subset grows exponentially with the number of variables, heuristic search methods are used to guide the search for an optimal subset. Guyon et al. [1] proposed one of the most popular wrapper approaches for variable selection in SVM. The method is known as SVM-Recursive Feature Elimination (SVM-RFE) and, when applied to a linear kernel, the algorithm is based on the steps shown in Fig. 1. The final output of this algorithm is a ranked list with variables ordered according to their relevance. In the same paper, the authors proposed an approximation for non-linear kernels. The idea is based on measuring the smallest change in the cost function by assuming no change in the value of the estimated parameters in the optimization problem. Thus, one avoids to retrain a classifier for every candidate variable to be eliminated.

SVM-RFE method is basically a backward elimination procedure. However, the variables that are top ranked (eliminated last) are not necessarily the ones that are individually most relevant but the most relevant conditional on the specific ranked subset in the model. Only taken together the variables of a subset are optimal in some sense. So for instance, if we are focusing on a variable that is *p* ranked we know that in the model with the 1 to *p* ranked variables, *p* is the variable least relevant.

The wrapper approaches include the interaction between variable subset search and model selection as well as the ability to take into account variable correlations. A common drawback of these techniques is that they have a higher risk of overfitting than filter methods and are computationally intensive, especially if building the classifier has a high computational cost [9]. Additional work has been done to assess variable importance in non-linear kernels SVM by modifying SVM-RFE [3, 10, 11].

The methods we propose in the next section are based on a wrapper approach, specifically in the RFE algorithm, allowing visualization and interpretation of the relevant variables in each RFE iteration using linear or non-linear kernels and fitting SVM extensions such as SVM for survival analysis,

## Methods

### RFE-pseudo-samples

One of our proposed methods follows and extends the idea proposed in Krooshof et al. [12] and Postma et al. [13] to visualize the importance of variables using pseudo-samples in the kernel partial least squares and the support vector regression (SVR) context, respectively. The proposed is applicable to SVM classifying binary outcomes. Briefly, the main steps are the following:

1. Optimize the SVM method and tune the parameters.
2. For each variable of interest, create a pseudo-samples matrix with equally distanced values *z*_∗_from the original variable, while maintaining the other variables set to their mean or median (1). *z*_*q*_ can be quantiles of the variable for an arbitrary *q* that is the number of selected quantiles. As the data is usually normalized, we assume that the mean is 0. There will be *p* pseudo-samples matrices of dimension *q x p*. For instance, for variable 1, the pseudo-sample matrix will look like in (1) with *q* pseudo-samples vectors.

1 $$ \left(\begin{array}{c}{V}_1\kern0.5em {V}_2\kern0.5em {V}_3\kern0.75em {V}_p\ \\ {}\begin{array}{ccc}{z}_1& 0& \begin{array}{cc}0& \begin{array}{cc}\dots & 0\end{array}\end{array}\end{array}\\ {}\begin{array}{ccc}{z}_2& 0& \begin{array}{cc}0& \begin{array}{cc}\dots & 0\end{array}\end{array}\end{array}\\ {}\begin{array}{ccc}{z}_3& 0& \begin{array}{cc}0& \begin{array}{cc}\dots & 0\end{array}\end{array}\end{array}\\ {}\vdots \\ {}\begin{array}{ccc}{z}_q& 0& \begin{array}{cc}0& \begin{array}{cc}\dots & 0\end{array}\end{array}\end{array}\end{array}\right)\ {\displaystyle \begin{array}{c}\begin{array}{c} pseudo- sample{s}_1\\ {} pseudo- sample{s}_2\\ {} pseudo- sample{s}_3\end{array}\\ {}\vdots \\ {} pseudo- sample{s}_q\end{array}} $$

3. Obtain the predicted decision value (not the predicted class) from SVM (a real negative or positive value) for each pseudo-sample using the SVM model fitted in step 1. Basically, this decision value corresponds to the distance of each observation from the SVM margins.
4. Measure the variability of each variable’s prediction using the univariate robust metric median absolute deviation (MAD). This mesure is expressed for a given variable *p* as

$$ MA{D}_p= median\left(|{D}_{qp}- median\left({D}_p\right)|\right)c $$

being *D*_*qp*_ the decision value of the variable *p* for the pseudo-sample *q* and being *median*(*D*_*p*_) the median of all decision values for the evaluated variable *p*. The constant *c* is equal to 1.4826, and it is incorporated in the expression to ensure consistency in terms of expectation so that

$$ E\left( MAD\left({D}_1,\dots, {D}_n\right)\right)=\sigma $$

for *D*_*i*_ distributed as *N*(*μ*, *σ*^2^) and large *n* [14, 15].

**Fig. 2:**
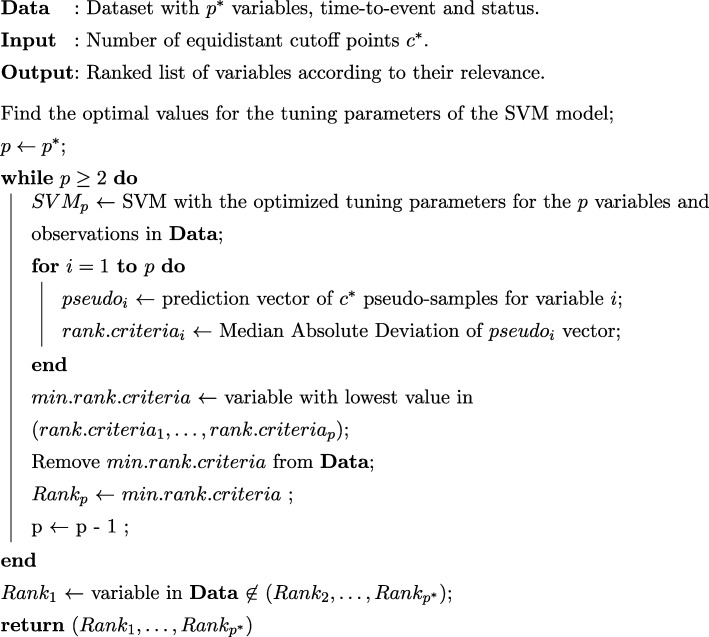
Pseudo-code of the RFE-pseudo-samples algorithm applied to a time-to-event (right-censored) response variable

5. Remove the variable with the lowest MAD value.
6. Repeat steps 2–5 until there is only one variable left (applying in this way the RFE algorithm as detailed in Fig. 2).

The rationale of the proposed method is that for variables associated with the response, modifications in the variable will affect predictions. On the contrary, for variables not associated with the response, changes in the variable value will not affect predictions and the decision value will be approximately constant. Therefore, since the decision value can be used as a score that measure distance to the hyperplane, the larger the absolute value the more confident we are that the observation belongs to the predicted class defined by the sign.

### Visualization of variables

The RFE-pseudo-samples algorithm allows us to plot the decision values and the range of all variables, in this way we account for:

- Strenght and direction of the association between individual variables and the response: since we are plotting the range of the variable and the decision value, we are able to detect whether larger values of the variable are protective or risk factors.
- The proposed method fix the values of the non-evaluated variables to 0 but this can be modified to evaluate the performance of the desired variables fixing the values to any other biologically meaningful value.
- The distribution of the data can be indicative of the type of association of each variable with respect the response, i.e., U-shaped, linear or exponential, for example.
- The variability on the decision values can be indicative of the relevance of the variable with the response. Given a variable, the more variability on the decision values along its range the more associated is the variable with the response.

### RFE-kernel principal components input variables

Reverter et al. [16] proposed a method using the kernel principal component analysis (KPCA) space (more detail on the KPCA methodology in Additional file 1) to represent, for each variable, the direction of maximum growth locally. So, given two leading components the maximum growth for each variable is indicated in a plot in which each axis is one of the components. After representing all observations in the new space, if a variable is relevant under this context will show a clear direction across all samples and if it’s not the sample’s direction will be random. In the same work the authors suggest to incorporate functions of the original variables into the KPCA space, so it’s possible to plot not only growth of individual variables but combination of them if makes sense within the research study. Our proposed method, referred as RFE-KPCA-maxgrowth, consists of the following steps:

1. Fit the SVM.
2. Create the KPCA space using the tuned parameters found in the SVM process with all variables if possible, for example, when the kernel used in SVM is the same than in KPCA.
3. Represent the observations with respect the two first components of the KPCA.
4. Compute and represent the input variables and the decision function of the SVM into the KPCA output, as detailed in Representation of input variables section.
5. Compute the average angle of each variable-observation with the decision function into the KPCA output. Therefore, an average angle using all observations, can be calculated for each variable (Ranking of variables section).
6. Calculate the difference for each variable between the average angle and the median of all variables average angle. The variable closest to the median is classified as the less relevant, as detailed in Ranking of variables section.
7. Remove the least relevant variable.
8. Repeat all the process from 1 to 7 until there is one variable left.

### Representation of input variables

We approach the problem of the interpretability of kernel methods by mapping simultaneously data points and relevant variables in a low dimensional linear manifold immersed in the kernel induced feature space *H* [17]. Such linear manifold, usually a plane, can be determined according to some statistical requirement, for instance, we shall require that the final Euclidean interdistances between points in the plot have to be, as far as possible, similar to the interdistances in the feature space, which shall lead us to the KPCA. We have to distinguish between the feature space *H* and the surface in that space to which points in input space *ℝ*^*p*^ actually map, which we denote by $\phi \left(\mathcal{X}\right)$. In general is a dimensional manifold embedded in *H*. We assume here that $\phi \left(\mathcal{X}\right)$ is sufficiently smooth that a Riemannian metric can be defined on it [18].

The intrinsic geometrical properties of $\phi \left(\mathcal{X}\right)$ can be derived once we know the Riemannian metric induced by the embedding of $\phi \left(\mathcal{X}\right)$ in *H*. The Riemannian metric can be defined by a symmetric metric tensor *g*_*ab*_. The explicit mapping to construct *g*_*ab*_ is unkonwn; it can be written solely in terms of the kernel [17].

Any relevant variable can be described by a real valued function *f* defined on the input space *ℝ*^*p*^. Since we assume that the feature map *ϕ* is one-to-one, we can identify *f* with $\overset{\sim }{f}\equiv f\circ {\phi}^{-1}$defined on $\phi \left(\mathcal{X}\right)$. We aim to represent the gradient of $\overset{\sim }{f}$. The gradient of $\overset{\sim }{f}$ is a vector field defined on $\phi \left(\mathcal{X}\right)$ through its components under the coordinates ***x*** = (*x*^1^, …, *x*^*p*^) as

2 $$ \operatorname{grad}{\left(\overset{\sim }{f}\right)}^a=\sum \limits_{b=1}^p{g}^{ab}\left(\boldsymbol{x}\right){D}_bf\left(\boldsymbol{x}\right)\ a=1,\dots, p $$

where *g*^*ab*^ is the inverse of the metric matrix *G* = (*g*_*ab*_) and *D*_*b*_ denotes the partial derivative with respect the *b* variable.

The curves *v* corresponding to the integral flow of the gradient, i.e., the curves whose tangent vectors at *t* are ${v}^{\prime }(t)=\mathit{\operatorname{grad}}\left(\overset{\sim }{f}\right)$. These curves indicate, locally, the maximum variation directions of $\widehat{f}$. Under the coordinates ***x*** = (*x*^1^, …, *x*^*p*^) the integral flow is the general solution of the first order differential equation system

3 $$ \frac{d{x}^a}{dt}=\sum \limits_{b=1}^p{g}^{ab}\left(\boldsymbol{x}\right){D}_bf\left(\boldsymbol{x}\right)\ a=1,\dots, p $$

which has always local solution given initial conditions *v*(*t*_0_) = ***w***.

To help interpreting the KPCA output, we can plot the projected *v*(*t*) curves (obtained in eq. 3) that indicates, locally, the maximum variation directions of $\overset{\sim }{f}$, or also, the corresponding gradient vector given in (2).

Let *v*(*t*) = *k*(∙, ***x***(*t*)) where ***x***(*t*) are the solutions of (3). If we define

4 $$ {\boldsymbol{Z}}_t={\left(k\left(\boldsymbol{x}(t),{\boldsymbol{x}}_i\right)\right)}_{n\mathrm{x}1}, $$

the induced curve, $\overset{\sim }{v}(t)$, expressed in matrix form, is given by the row vector

5 $$ \overset{\sim }{v}{(t)}_{1\mathrm{x}r}^q=\left({\boldsymbol{Z}}_t^{\prime }-\frac{1}{n}{\mathbf{1}}_n^{\prime }K\right)\left({\boldsymbol{I}}_n-\frac{1}{n}{\mathbf{1}}_n{\mathbf{1}}_n^{\prime}\right)\overset{\sim }{\boldsymbol{V}} $$

where ***Z***_*t*_ has the form (4), and ^′^ symbol indicates transposed.

We can also represent the gradient vector field of $\widehat{f}$, that is, the tangent vector field corresponding to curve *v*(*t*) through its projection into the KPCA output. The tangent vector at *t* = *t*_0_, if *x*_0_ = *ϕ*^−1^ ∘ *v*(*t*_0_) is given by${\left.\frac{dv}{dt}\right|}_{t={t}_0}$, and its projection, in matrix form, is given by the row vector

6 $$ {\left({\left.\frac{d\overset{\sim }{v}}{\mathrm{d}t}\right|}_{t={t}_0}\right)}_{1\mathrm{x}r}={\left.\frac{d{\boldsymbol{Z}}_t^{\prime }}{dt}\right|}_{t={t}_0}\left({\boldsymbol{I}}_n-\frac{1}{n}{\mathbf{1}}_n{\mathbf{1}}_n^{\prime}\right)\overset{\sim }{\boldsymbol{V}} $$

with

7 $$ {\left.\frac{d{\boldsymbol{Z}}_t^{\prime }}{dt}\right|}_{t={t}_0}={\left({\left.\frac{d{\boldsymbol{Z}}_t^1}{dt}\right|}_{t={t}_0},\dots, {\left.\frac{d{\boldsymbol{Z}}_t^n}{dt}\right|}_{t={t}_0}\right)}^{\prime }, $$

and,

8 $$ {\left.\frac{d{\boldsymbol{Z}}_t^i}{dt}\right|}_{t={t}_0}={\left.\frac{dk\left(\boldsymbol{x}(t),{\boldsymbol{x}}_i\right)}{dt}\right|}_{t={t}_0}=\sum \limits_{a=1}^p{D}_ak\left({\boldsymbol{x}}_0,{\boldsymbol{x}}_i\right){\left.\frac{d{x}^a}{dt}\right|}_{t={t}_0} $$

where ${\left.\frac{d{x}^a}{dt}\right|}_{t={t}_0}$is defined in (3).

### Ranking of variables

Our proposal is to take advantage of the representation of direction of input variables applying two alternative approaches:

- To include the SVM predicted decision values for each training sample as an extra variable, what we call *reference variable*. Then, compare directions of each one of the input variables with the reference.
- To include the direction of the SVM decision function and use it as the *reference direction*. Since it is as a real-valued function of the original variables we can represent the direction of this expression. Specifically, the decision function removing the sign function of the expression of SVM is given by

9 $$ f\left(\boldsymbol{x}\right)=\sum \limits_{i=1}^n{\alpha}_i{y}_ik\left({\boldsymbol{x}}_i,\boldsymbol{x}\right)+b $$

we can reformulate (9) to

10 $$ f\left(\boldsymbol{x}\right)=\sum \limits_{i=1}^n{\varrho}_ik\left({\boldsymbol{x}}_i,\boldsymbol{x}\right)+b $$

where *ϱ*_*i*_ = *α*_*i*_*y*_*i*_. Applying the representation of input variables methodology to function (10) and assuming Gaussian kernel expressed as $k\left({\boldsymbol{x}}_1,{\boldsymbol{x}}_2\right)=\exp \left(-\frac{1}{\sigma }{\left\Vert {\boldsymbol{x}}_1-{\boldsymbol{x}}_2\right\Vert}^2\right)$, from formula (8),

we obtain

$$ {\left.\frac{d{\boldsymbol{Z}}_t^i}{dt}\right|}_{t=0}=k\left({\boldsymbol{x}}_i,\boldsymbol{x}\right)\sum \limits_{a=1}^p\left({x}_i^a-{x}^a\right)\left[\sum \limits_{j=1}^n{\varrho}_i\ \sigma \left({x}_j^a-{x}^a\right)k\left({\boldsymbol{x}}_j,\boldsymbol{x}\right)\right] $$

**Fig. 3:**
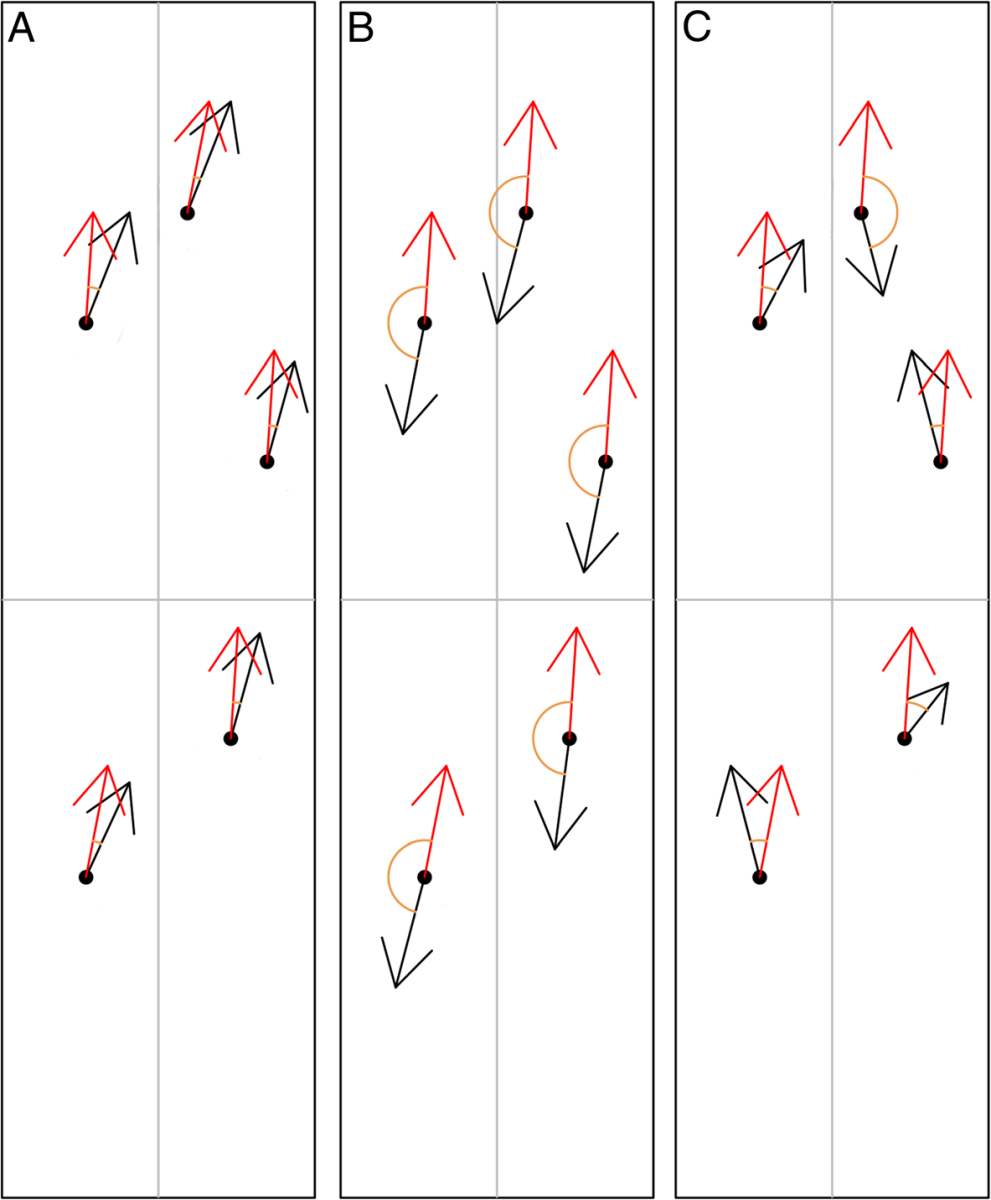
Visual representation of variable importance. Vectors are the projection on the two leading KPCA axes of the vectors in the kernel feature space pointing to the direction of maximum locally growth of the represented variables. In this scheme, the reference variable is in red and original variables are in black. Each sample point anchors a vector representing the direction of maximum locally growth. **a** When an original variable is associated with the reference variable, the angle between both vectors, averaged across all samples, is close to zero radians. **b** In contrast, when an original variable is negatively associated with the reference variable, the angle between both vectors, averaged across all samples, is close to *π* radians. **c** When an original variable does not show any association with the reference variable, the angle changes non-consistently among the samples. In noisy data, behavior (**c**) is expected to occur in most variables, so the variable with average angle closest to the overall angle after accounting for all variables is assumed to be the least relevant

**Fig. 4:**
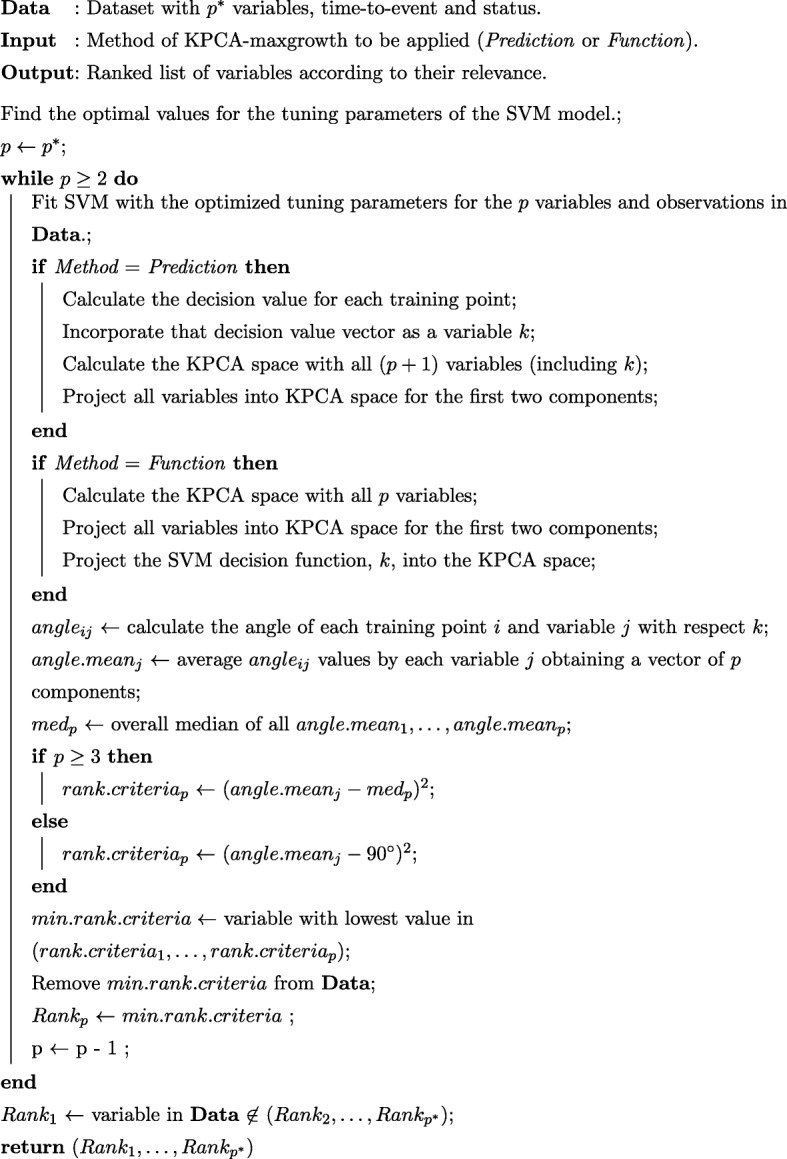
Pseudo-code of the RFE-KPCA-maximum-growth algorithm for both function and prediction approach. The algorithm is applied to a time-to-event (right-censored) response variable

For both prediction values and decision function, we can calculate the overall similarity of one variable with respect the reference (either the prediction or the decision function) by averaging the angle of the maximum growth vector for all training points with the reference. So, if, for a given training point, the angle of the direction of maximum growth of variable *p* with the reference is 0 (0 rad) would mean that the vector of directions overlap and they are perfectly positively associated. If the angle is 180 (*π* radians) they go in opposite direction, indicating that they are perfectly negatively associated (Fig. 3). By averaging the angle of all training points we obtain a summary of the similarity of each variable with the reference and, consequently, whether is relevant or not. Assuming that there is noise in real data, a variable is classified as relevant or not compared to the others: the variable closest to the overall angle taking into account all variables is assumed to be the least relevant. Based on this, we can apply a RFE-KPCA-maximum-growth approach for prediction and for decision function as defined by Fig. 4.

### Visualization of importance of variables

We can represent for each observation the original variables as vectors (with a pre-specified length), that indicate the direction of maximum growth in each variable or a function of each variable. When two variables are positively correlated, the directions of maximum growth for all samples should appear in the same direction and in the perfect scenario samples should overlap. When two variables are negatively correlated the direction should be overall opposite, i.e., should be a mirror image, and if they are no correlated, directions should be random (Fig. 3).

### Compared scenarios

To fix ideas, we applied the three proposed approaches: RFE-pseudo-samples, RFE-KPCA-maxgrowth-prediction and RFE-KPCA-maxgrowth-decision and compared them to the RFE-Guyon for non-linear kernels. These methods are applied to analyse simulated and real time-to-event data with SVM. We simulated a time-to-event response variable and the corresponding censoring distribution. To evaluate the performance of the proposed methods in this survival framework, several scenarios involving different correlated variables have been simulated.

### Simulation of scenarios and data generation

We generated 100 datasets with a time-to-event response variable and 30 predictor variables following a multivariate normal distribution. The mean of each variable was a realization of a Uniform distribution U(0.03,0.06) and the covariance matrix was computed so that all variables were classified in four groups according to their pairwise correlation: no correlation (around 0), low correlation (around 0.2), medium correlation (around 0.5) and high correlation (around 0.8). The variance distribution of each variable was fixed to 0.7 (see correlation matrix at Additional File 2).

The time-to-event variable was simulated based on the proportional hazards assumption through a Gompertz distribution [19]:

11 $$ T=\frac{1}{\alpha}\left(1-\frac{\alpha \log (U)}{\gamma \exp \left(\left\langle \boldsymbol{\beta}, {\boldsymbol{x}}_i\right\rangle\ \right)}\right) $$

where *U* is a variable following a Uniform(0,1) distribution, ***β*** is the coefficients variable vector, *α* ∈ (−∞, ∞) and *γ*> 0 are the scale and shape parameters of the Gompertz distribution. These parameters were selected so that overall survival was around 0.6 at 18 months follow-up time.

The number of observations in each dataset was 50 and the time of censoring distribution followed a Uniform allowing around 10% censoring.

### Relevance of variables scenarios

To evaluate the proposed methods, we generated the time-to-event response variable assuming the following scenarios: i) large and low pairwise correlation among predictors, some of them with variables highly associated with the response and others not, ii) positive and negative association with the response variable, and iii) linear and non-linear associations with the response variable and, in some cases, interaction among predictor variables. The relevant variables for each one of the 6 simulated scenarios are:

1. Variable 1.
-Variable 29 + Variable 30.
-Variable 1 + Variable 8 + Variable 20 + Variable 29 - Variable 30.
4. Variable 1 + Variable 2 + Variable 1 x Variable 2.
5. Variable 1 + Variable 30 + Variable 1 x Variable 30 + Variable 20 + (Variable 20)^2^.
6. Variable 1 + (Variable 1)^2^ + exp(Variable 30).

### Real-life datasets

The PBC, Lung and DLBCL datasets freely available at the CRAN repository were used as real data to test the performance of the proposed methods. Briefly, datasets of the following studies were analyzed:

- PBC: this data is from the Mayo Clinic trial in primary biliary cirrhosis of the liver conducted between 1974 and 1984. The study aimed to evaluate the performance of the drug D-penicillamine in a placebo controlled randomized trial. This data contains 258 observations and 22 variables (17 of them are predictors). From the whole cohort 93 observations experienced the event, 65 finalized the follow-up period being a non-event, and thus were censored, and 100 were censored before the end of the follow-up time of 2771 days, with an overall survival probability of 0.57.
- Lung: this study was conducted by the North Central Cancer Treatment Group (NCCTG) and aimed to estimate the survival of patients with advanced lung cancer. The available dataset included 167 observations, experiencing 89 events during the follow-up time of 420 days, and 10 variables. A total of 36 observations were censored before the end of follow-up. The overall survival was 0.40.
- DLBCL: this dataset contains gene expression data from diffuse large B-cell lymphoma (DLBCL) patients. The available dataset contains 40 observations and 10 variables representing the mean gene expression in 10 different clusters. From the analysed cohort 20 patients experienced the event, 10 finalized the follow-up and 8 were right-censored during the 72 months follow-up period.

Cox proportional-hazards models were used and compared with the proposed methods. We applied the RFE algorithm and in each iteration the variable with lowest proportion of explainable log-likelihood in the Cox model was removed. To compare the obtained rank of variables the correlation between the ranks was computed. Additionally, the C statistic was computed by ranked variable and method to evaluate its discriminative ability.

### Probabilistic SVM

The data was analysed with a modified SVM for survival analysis that was previously considered optimal to handle censored data [20]. The method, known as probabilistic SVM [21] (more details on this method on Additional file 3), allows not perfectly defining some observations and give them an uncertainty in their class. For these uncertainties a confidence level or probability regarding the class is provided.

### Comparison of methods

The parameters selected to perform the grid-search for Gaussian kernel were 0.25, 0.5, 1, 2 and 4. The *C* and $\overset{\sim }{C}$ values were 0.1, 1, 10 and 100. For each combination of parameters, a tunning parameter step with 10 training datasets were fitted and validated using 10 different validation datasets. Additionally, 10 training datasets, different from all datasets used in the tuning parameters step, were simulated and fitted with the best combination found in tuning parameters step. The tuned parameters were fixed for each RFE iteration, i.e., were not estimated at each iteration. Once the optimal parameters for the pSVM were found the methods compared were:

- RFE-Guyon for non-linear data: this method was considered the gold standard.
- RFE-KPCA-maxgrowth-prediction: the KPCA is based on Gaussian kernel with parameters obtained in the pSVM model.
- RFE-KPCA-maxgrowth-decision: the KPCA is based on Gaussian kernel with parameters obtained in the pSVM model.
- RFE-pseudo-samples: the range of the data, to create the pseudo-samples is created split- ting data into 50 equidistant points. The range of the pseudo-samples goes from − 2 to 2, since variables are normally distributed around 0 approximately.

### Metrics to evaluate algorithm performance

The mean and standard deviation of the rank obtained in 100 simulated datasets was used to summarize the performance by method and scenario. For the RFE-pseudo-samples algorithm the first iteration figure with all 100 datasets was created summarizing the information by variable. For the RFE-maxgrowth approach, as example, one of the datasets was presented in order to interpret the method, since it was not possible to summarize all 100 principal components plots in one figure.

## Results

### Simulated datasets

In this section, main results are described by algorithm and scenario. Results are structured according to overall ranking of variables and visualization and interpretation of two scenarios for illustrative purposes.

### Overall ranking comparison

**Fig. 5:**
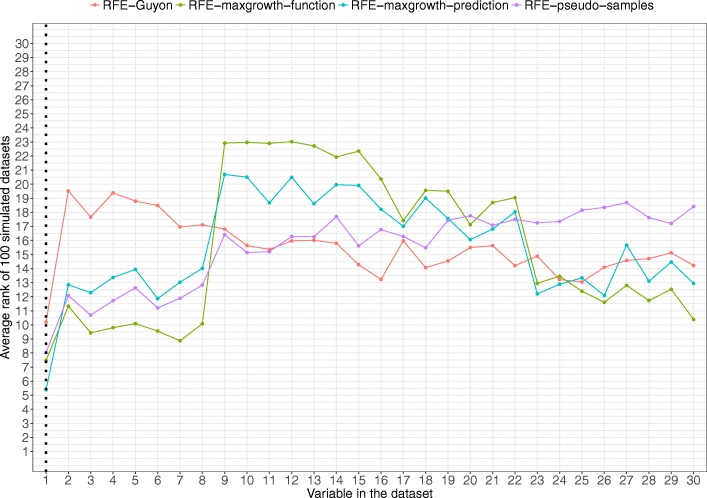
Scenario 1 results. Average rank by variable and method for the 100 simulated datasets for Scenario 1 (being Variable 1 the relevant variable). Dotted vertical black line represents the variable used to generate the time-to-event variable. The lower the rank, the more relevant the variable is for the specific algorithm

Scenario 1 results are shown in Fig. 5. All 4 methods identified the relevant variable being the RFE-maxgrowth-prediction the one with the lowest average rank (thus, optimal), followed by the RFE-maxgrowth-function, RFE-pseudo-samples and RFE-Guyon. For all methods, except the RFE- Guyon, a set of variables was closest to the Variable 1 rank (variables 2 to 8). These variables were highly correlated with Variable 1.

**Fig. 6:**
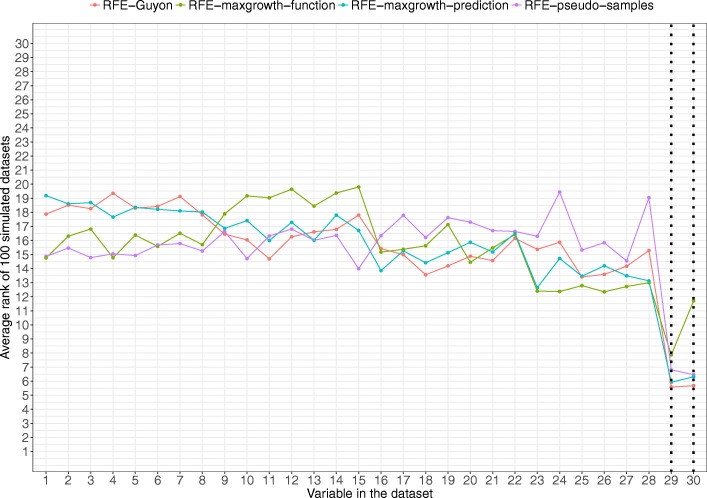
Scenario 2 results. Average rank by variable and method for the 100 simulated datasets for Scenario 2 (being variables 29 and 30 the relevant variables). Dotted vertical black lines represent the variable used to generate the time-to-event variable. The lower the rank, the more relevant the variable is for the specific algorithm

For scenario 2 (Fig. 6), the true relevant variables were identified for all 4 algorithms, being the average rank pretty similar, except the RFE-maxgrowth-function. The specific overall rank order was RFE-Guyon, RFE-maxgrowth-prediction, RFE-pseudo-samples and RFE-maxgrowth-function. The average rank for the other non-relevant variables was similar for all methods. In this scenario the relevant variables were not correlated with any other variable in the dataset.

**Fig. 7:**
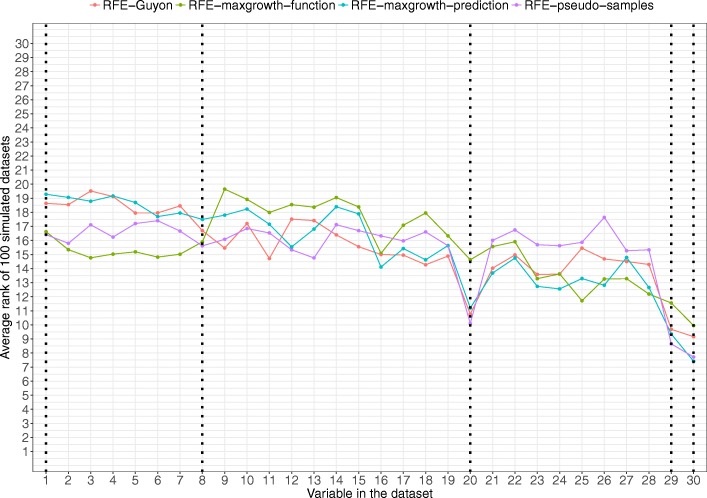
Scenario 3 results. Average rank by variable and method for the 100 simulated datasets for Scenario 3 (being variables 1, 8, 20, 29 and 30 the relevant variables). Dotted vertical black lines represent the variables used to generate the time-to-event variable. The lower the rank, the more relevant the variable is for the specific algorithm

In scenario 3 (Fig. 7), 5 variables are relevant in the true model. The algorithms were able to detect the relevant non-correlatedvariables (variables 20, 29 and 30), except the RFE-maxgrowth-function, that for this set of variables was the worst method. For the other 3 algorithms and this set of variables, the RFE-pseudo-samples was slightly better and the RFE-Guyon slightly worst than the others. For the other 2 highly correlated variables (Variable 1 and Variable 8) the two best methods were clearly RFE-pseudo-samples and RFE-maxgrowth-function.

**Fig. 8:**
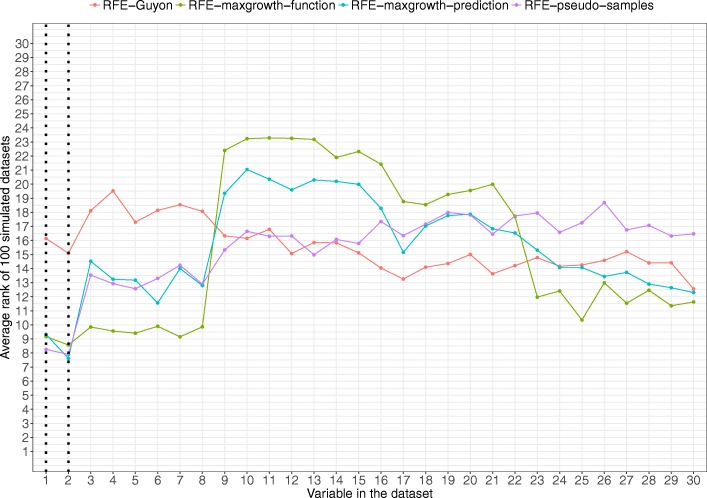
Scenario 4 results. Average rank by variable and method for the 100 simulated datasets for Scenario 4 (being variables 1 and 2 the relevant variables). Dotted vertical black lines represent the variables used to generate the time-to-event variable. The lower the rank the more relevant the variable is for the specific algorithm

In Scenario 4 (Fig. 8), all methods, except RFE-Guyon, detected the two relevant variables. However, RFE-maxgrowth-function identified as relevant, with a pretty similar rank, variables 3 to 8 (highly correlated with the true relevant ones). The RFE-pseudo-samples algorithm ranks increased as the correlation with the true relevant variables decreased.

**Fig. 9:**
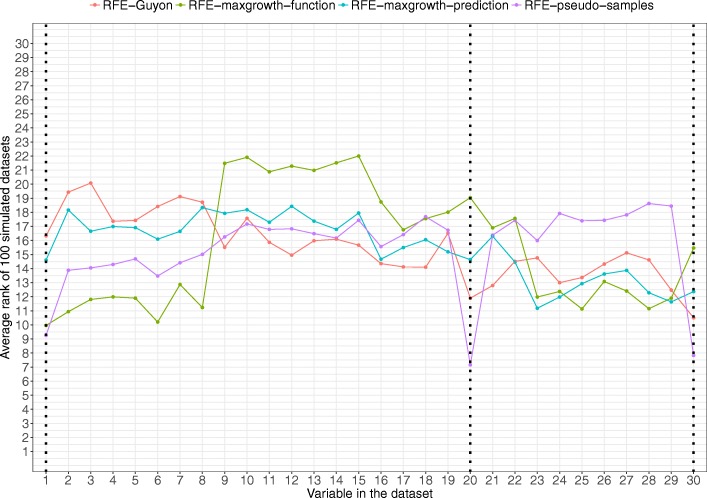
Scenario 5 results. Average rank by variable and method for the 100 simulated datasets for Scenario 5 (being variables 1, 20 and 30 the relevant variables). Dotted vertical black lines represent the variable used to generate the time-to-event variable. The lower the rank the more relevant the variable is for the specific algorithm

For Scenario 5 (Fig. 9) three variables were relevant (1, 20 and 30). An interaction and a quadratic term were included. RFE-pseudo-samples was clearly the method that best identified the relevant variables. The other three algorithms were not able to detect the three variables, although RFE-maxgrowth-function was able to identify as relevant, with a similar rank, variables 1 to 8 (highly correlated among them).

**Fig. 10:**
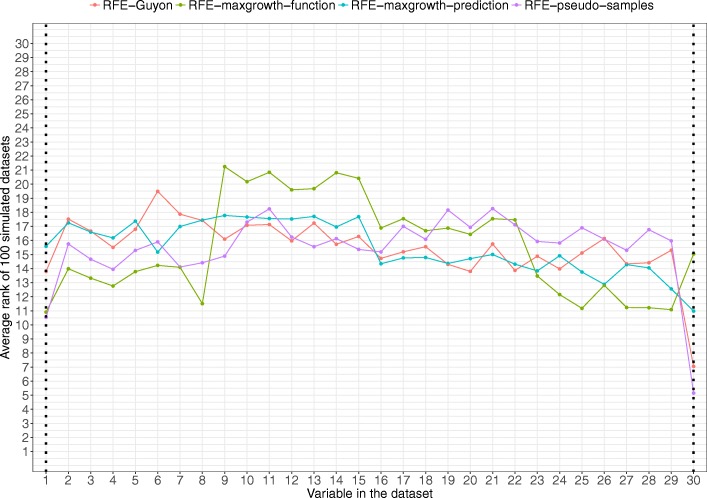
Scenario 6 results. Average rank by variable and method for the 100 simulated datasets for Scenario 6 (being variables 1 and 30 the relevant variables). Dotted vertical black lines represent the variable used to generate the time-to-event variable. The lower the rank the more relevant the variable is for the specific algorithm

In Scenario 6 (Fig. 10), Variable 1 and Variable 30 were selected as relevant; being the former included as main effect with a quadratic term and the latter exponentiated. All methods, except RFE-maxgrowth-function, were able to detect the importance of Variable 30. With respect to Variable 1, RFE-pseudo-samples and RFE-Maxgrowth-function yielded a similar rank of approximately 10.5. The other two algorithms, RFE-Guyon and RFE-maxgrowth-prediction, were not able to identify as relevant Variable 1 with the ranks for this variable comparable to to other non-relevant variables.

### Visualization of proposed methods

#### RFE-pseudo-samples

**Fig. 11:**
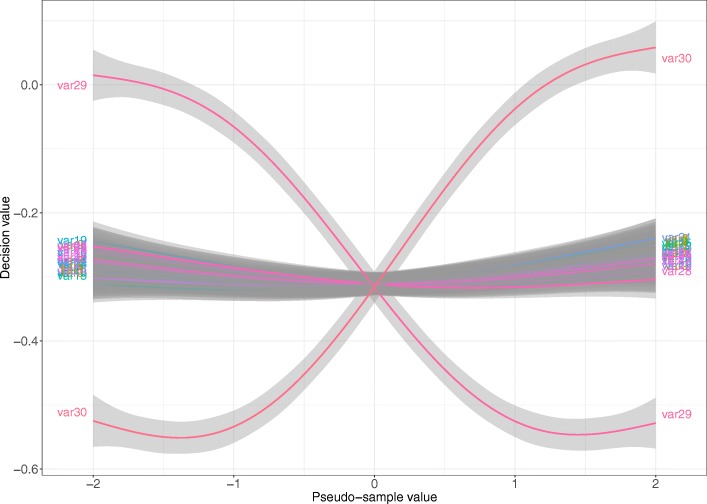
Visualization of RFE-pseudo-samples results for Scenario 2. Results for Scenario 2 (in which variables 29 and 30 were the relevant variables) over all 100 simulated datasets, all 30 variables, and first iteration of the RFE-pseudo-samples algorithm. The pseudo-samples distribution for each variable is shown with a non-parametric local regression estimation (LOESS) with the corresponding 95% confidence interval

An example of the results for Scenario 2 (all other scenarios are included as Additional files, from Additional Files 4, 5, 6, 7, 8 and 9), the 100 simulated datasets and first iteration of the RFE algorithm is shown in Fig. 11. Two variables show a completely different pattern from the others: Variable 29 and Variable 30. The association with the response of them was a mirror image of each other: for Variable 30, the larger the pseudo-sample value the larger the decision value and for Variable 29, the larger the pseudo-sample the lower the decision value. The other variables are pretty constant along the pseudo-samples range.

#### RFE-KPCA-maxgrowth prediction and function

**Fig. 12:**
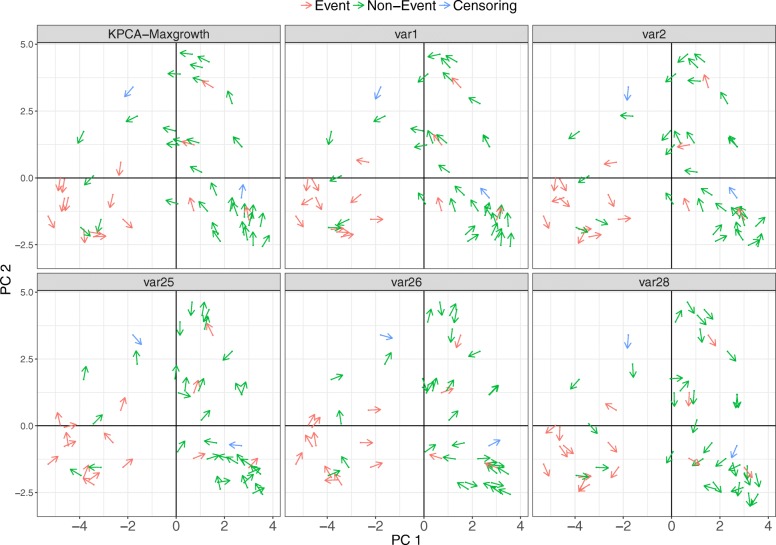
Visualization of RFE-KPCA-maxgrowth results for Scenario 1. Scenario 1 (being Variable 1 the relevant variable) results for a random simulated dataset and iteration 25 of the RFE-KPCA-maxgrowth-prediction approach. The first component of the KPCA (PC1) is represented in the X-axis and the second component (PC2) is represented in the Y-axis. Events, non-events (censored at the end of follow-up time) and losses to follow-up (censored during follow-up) are represented by red, green and blue color, respectively

Figure 12 shows an example of RFE-maxgrowth-prediction algorithm, Scenario 1, and iteration 25. To make the plot more interpretable, we only displayed the 5 variables selected as the most relevant: 1, 2, 25, 26 and 28. The first two were highly correlated (in average, a 0.8 Pearson correlation) and the others were independent by design. The reference is the prediction approach, but it is equivalent to function approach. The first component (PC1) is the one that classifies the event group, most events are negative and non-events are positive. For the reference, the directions are going from non-event to event along the PC1 and PC2. With respect to the other variables, only Variable 1 and Variable 2 present a pattern in terms of directions for each observation similar to the reference. Variables 25, 26 and 28 look pretty random. The interpretation of this is: variables 1, 2 and the reference perform similarly, thus, Variable 1 and Variable 2 are relevant and the others are not. Besides that, since 25, 26 and 28 directions are random between them, they are not associated with the response and they are not correlated, which is true by the data generation mechanism.

#### Real-life datasets

**Fig. 13:**
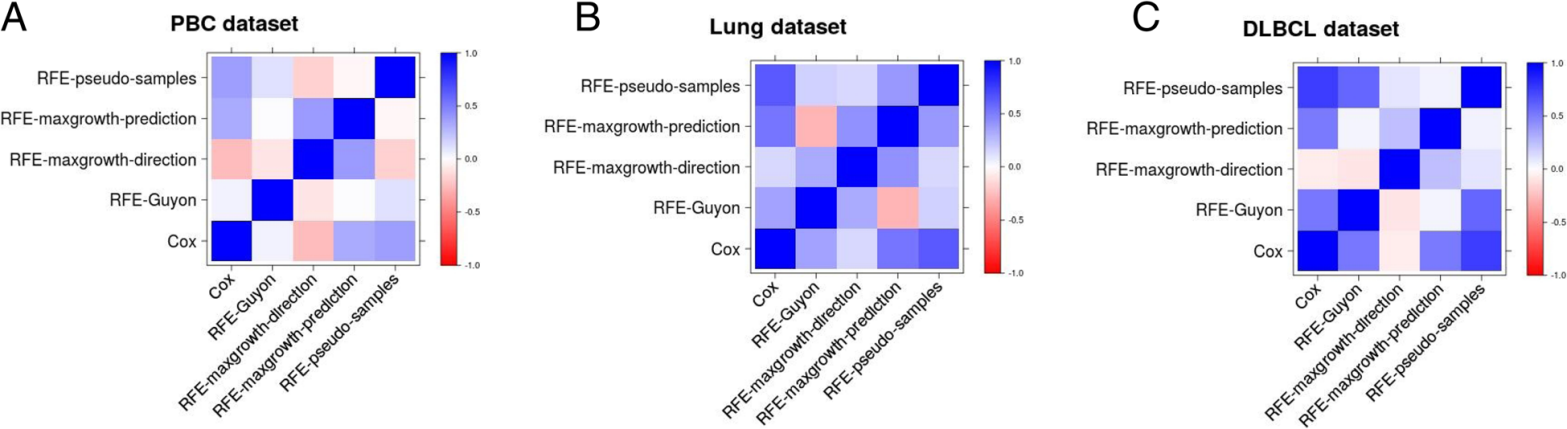
Spearman correlation matrix comparing 5 methods in the **a** PBC, **b** Lung and **c** DLBCL datasets. The Spearman correlation was computed comparing the ranks obtained by each one of the variables in the dataset

In Fig. 13 the Spearman correlation between each method comparing the obtained ranks for each one of the variables in the three dataset is shown. In all three compared real datasets the RFE-pseudo-samples and RFE-maxgrowth-prediction were the methods most correlated with the Cox model. In the Additional Files 10, 11 and 12, the rank comparison between each method and PBC, DLBCL and Lung datasets, respectively, is presented.

**Fig. 14:**
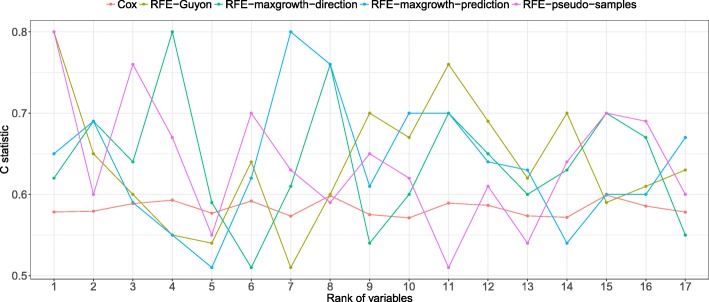
Discriminative ability, measured as C statistic, by method and ranked variable in the PBC dataset. The X-axis shows the rank of each one of the variables in the dataset after applying the RFE algorithm. The lower the rank the more relevant the variable is and the larger the C statistic is expected. As each method can rank differently the variables, given a rank the variable can be different between methods, due to this the C statistic (Y-axis) is different

**Fig. 15:**
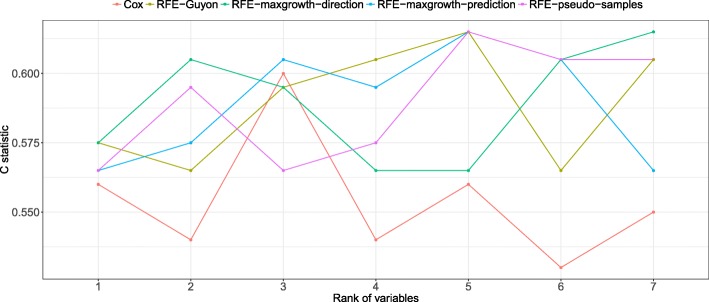
C statistics results by method and ranked variable in the Lung dataset. The X-axis shows the rank of each one of the variables in the dataset after applying the RFE algorithm. The lower the rank the more relevant the variable is and the larger the C statistic is expected. As each method can rank differently the variables, given a rank the variable can be different between methods, due to this the C statistic (Y-axis) is different

**Fig. 16:**
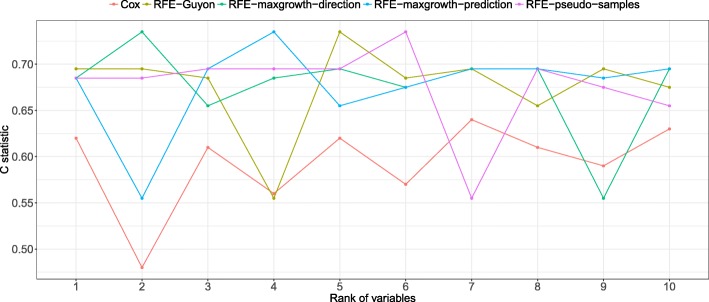
C statistics results by method and ranked variable in the DLBCL dataset. The X-axis shows the rank of each one of the variables in the dataset after applying the RFE algorithm. The lower the rank the more relevant the variable is and the larger the C statistic is expected. As each method can rank differently the variables, given a rank the variable can be different between methods, due to this the C statistic (Y-axis) is different

From Figs. 14, 15 and 16 the C statistic results by method and real dataset are shown. The RFE-pseudo-samples method discriminative ability is better than the other ones, especially in the DLBCL and PBC dataset, were the C statistics of the top ranked variables (the ones classified by the algorithm as more relevants) are larger. The RFE-maxgrowth methods perform slightly better than the RFE-Guyon except in DLBCL dataset (Fig. 16) were RFE-Guyon performance is overall better being the C statistic better in larger ranks.

## Discussion

In biomedical research, it is important to select the variables most associated with the studied outcome and to learn about the strength of this association. In SVM with non-linear kernels, variable selection is particularly challenging because the feature and input spaces are different, thus learning about variables in the feature space does not address the main question about variables in the original space. Although non-linear kernels, specially the Gaussian kernel, are widely used, little work has been done comparing methods to select variables in SVM with non-linear kernels. Moreover, almost no work has focused on interpretation and visualization of the association predictor-response in SVM with linear or non-linear kernels to help the analyst to not only select variables but also learn about the strength and direction of the association. The algorithms we proposed here for SVM aimed to fill this gap and allow analysts to use SVM to better address common scientific questions, i.e.: select variables when using non-linear kernels and learn about the strength of associations of predictor-response. Moreover, the algorithms presented are applicable for analysis of time-to-event responses that are often the primary outcomes in biomedical research.

The three algorithms we proposed performed generally better than the gold standard RFE-Guyon for non-linear kernels. As expected, results for all methods were better when the true relevant variables were independent, i.e., they were no correlated with the other variables in the SVM model. However, this scenario is rarely the case in biomedical research, particularly when analysis includes several variables. Generally, the RFE-pseudo-samples outperformed the other three methods in all tested scenarios. Additionally, the RFE-pseudo-samples algorithm rendered a more friendly visualization of results than RFE-Guyon.

With regards to the RFE-maxgrowth, both prediction and function approaches performed similarly. The prediction approach identified the relevant variables better than the fuction approach and the function was less time consuming. The prediction approach can be interpreted as an instance of the function. Although the RFE-maxgrowth-function was based on the explicit decision function and, thus, was expected to outperform the other three approaches, it did not perform as accurately as the other three approaches. One explanation could be that by approaching the decision function with a non-linear kernel as a combination of variables we are loosing more information than by using the RFE-maxgrowth-prediction.

In the RFE-maxgrowth-prediction algorithm, the prediction was included as an extra variable into the KPCA space. When including this extra variable, the constructed space accounts for the patterns that define event and non-event into the KPCA and is different from the constructed space ignoring the prediction variable. However, in the RFE-maxgrowth-function the KPCA space does not take into account any specific variable directly related to the classes.

The interpretation of the RFE-maxgrowth algorithm is more complex than the RFE-pseudo-samples algorithm because it includes interpretation of the components of the KPCA, the directions of maximum growth of each input variable, and the comparison of the direction of the maximum growth of the input variables between the event and non-events. Although this approach is more informative, it can only be interpreted for a reduced number of variables.

When analyzing the three real datasets the three SVM methods performed overall better than Cox model which is the classical statistical model to analyze time-to-event data. Moreover, the three real datasets fit in terms of sample size and number of variables into the Cox assumptions. Within the proposed methods the RFE-pseudo-samples performed better than the others, being the top-ranked variables the ones with largest discriminative power. The RFE-maxgrowth methods performed slightly better than RFE-Guyon. The obtained results in the real datasets are consistent with the ones obtained in the simulation study.

The main limitation of the proposed methods is that they are more computationally intensive than classical RFE-Guyon. That could be a limitation depending on the size of the database, the proportion of censored observations during the follow-up period or the SVM extension model used to analyze the time-to-event data. However, this shouldn’t be an extra complexity point when analyzing binary response data with no censored obsevations.

Further extensions of the presented work are the comparisons of the proposed methods with other machine learning agorithms used to identify relevant variables such as Random Forest, Elastic Net or Correlation-based Feature Selection evaluator, by analyzing simulated scenarios and real datasets. Additionaly, future work should focus in another important part of the identification of relevant features which is finding the method with largest accuracy or discriminatory ability and not only the identification of the true relevant variables.

## Conclusion

Conducting variable selection and interpreting associations between predictors and response variables with the proposed approaches when analyzing biomedical data using SVM with non-linear kernels has some advantadges over the currently available RFE of Guyon. Additionally, the proposed approaches can be implemented with high level of accuracy and speed, and with low computational cost, particularly when using the RFE-pseudo-samples algorithm. Although the proposed methods had more difficulties to identify relevant variables when those variables were highly correlated, they performed better than the classical RFE algorithm with non-linear kernels proposed by Guyon.

## Additional files

Additional file 1: Kernel feature space and kernel principal component analysis methodology. (DOCX 42 kb)

Additional file 2: Pearson correlation matrix of the 30 variables simulated. (PDF 131 kb)

Additional file 3: Probabilistic support vector machine methodology. (DOCX 36 kb)

Additional file 4: Visualization of RFE-pseudo-samples results for Scenario 1. Scenario 1 (being Variable 1 the relevant variable) results for all 100 simulated datasets, all 30 variables and first iteration of the RFE-pseudo-samples algorithm. The pseudo-samples distribution for each variable is shown with a non-parametric local regression estimation (LOESS) with the corresponding 95% confidence interval. (PDF 61 kb)

Additional file 5: Visualization of RFE-pseudo-samples results for Scenario 2. Scenario 2 (being Variable 29 and 30 the relevant variables) results for all 100 simulated datasets, all 30 variables and first iteration of the RFE-pseudo-samples algorithm. The pseudo-samples distribution for each variable is shown with a non-parametric local regression estimation (LOESS) with the corresponding 95% confidence interval. (PDF 59 kb)

Additional file 6: Visualization of RFE-pseudo-samples results for Scenario 3. Scenario 3 (being Variable 1, 8, 20, 29 and 30 the relevant variables) results for all 100 simulated datasets, all 30 variables and first iteration of the RFE-pseudo-samples algorithm. The pseudo-samples distribution for each variable is shown with a non-parametric local regression estimation (LOESS) with the corresponding 95% confidence interval. (PDF 57 kb)

Additional file 7: Visualization of RFE-pseudo-samples results for Scenario 4. Scenario 4 (being Variable 1 and 2 the relevant variables) results for all 100 simulated datasets, all 30 variables and first iteration of the RFE-pseudo-samples algorithm. The pseudo-samples distribution for each variable is shown with a non-parametric local regression estimation (LOESS) with the corresponding 95% confidence interval. (PDF 61 kb)

Additional file 8: Visualization of RFE-pseudo-samples results for Scenario 5. Scenario 5 (being Variable 1, 20 and 30 the relevant variables) results for all 100 simulated datasets, all 30 variables and first iteration of the RFE-pseudo-samples algorithm. The pseudo-samples distribution for each variable is shown with a non-parametric local regression estimation (LOESS) with the corresponding 95% confidence interval. (PDF 53 kb)

Additional file 9: Visualization of RFE-pseudo-samples results for Scenario 6. Scenario 6 (being Variable 1 and 30 the relevant variables) results for all 100 simulated datasets, all 30 variables and first iteration of the RFE-pseudo-samples algorithm. The pseudo-samples distribution for each variable is shown with a non-parametric local regression estimation (LOESS) with the corresponding 95% confidence interval. (PDF 60 kb)

Additional file 10: Results for PBC dataset comparing the four RFE algorithms and the Cox model. (PDF 6 kb)

Additional file 11: Results for DLBCL dataset comparing the four RFE algorithms and the Cox model. (PDF 5 kb)

Additional file 12: Results for Lung dataset comparing the four RFE algorithms and the Cox model. (PDF 5 kb)

## Abbreviations

KPCA: Kernel principal component analysis
RFE: Recursive Feature Elimination
SVM: Support vector machines
