# Supplementary material for: SVM-RFE: selection and visualization of the most relevant features through non-linear kernels
Source: BMC Bioinformatics. 2018 Nov 19;19:432. doi: 10.1186/s12859-018-2451-4 (PMC6245920; doi:10.1186/s12859-018-2451-4)
Supplement: Supplementary file 1 — Kernel feature space and kernel principal component analysis methodology. (DOCX 42 kb) [file 12859_2018_2451_MOESM1_ESM.docx]

Kernel feature space

Given a sample space $\mathcal{X}$, a real valued positive kernel $k$ on $\mathcal{X}$ is a map $k:\mathcal{X} \text{x }\mathcal{X}\mathbb{\to R}$ such that $k\left( \boldsymbol{x},\boldsymbol{y} \right)=k\left( \boldsymbol{y},\boldsymbol{x} \right)=\sum_{i,j}^{n} \alpha_{i}\alpha_{j}k\left( x_{i},x_{j} \right)\geq0$ for all $n\mathbb{\in N}$, $\alpha_{i}\mathbb{\in R}$, $x_{i}\in\mathcal{X} i=1,\ldots,n$, and the equality is attained if and only if all the coefficients $\alpha_{i}$are equal to zero. A kernel can be interpreted as a similarity measure of the samples and allow us to identify, in a natural way, each $\boldsymbol{x}\in\mathcal{X}$ with a real valued function, which contains all relevant features of the sample point $\boldsymbol{x}$, given by

$$\phi:\mathcal{X \to}R^{\mathcal{X}} \equiv\{f:\mathcal{X}\mathbb{\to R\}}$$

$$\boldsymbol{x} \mapsto\phi\left( \boldsymbol{x} \right):=k(\cdot,\boldsymbol{x})$$

this function is an element of a dot product vector space that will be called kernel feature space[20] It consists of all real valued functions $f$ which can be expressed as $f\left( \cdot\right)= \sum_{i=1}^{n} \alpha_{i}k(\cdot,\boldsymbol{x}_{\boldsymbol{i}})$ for any $n\mathbb{\in N}$, $x_{1},\ldots,x_{n}\in\mathcal{X} , \alpha_{1},\ldots, \alpha_{n}\mathbb{\in R.}$ Given $f\left( \cdot\right)= \sum_{i=1}^{n} \alpha_{i}k(\cdot,\boldsymbol{x}_{\boldsymbol{i}})$ and $g\left( \cdot\right)= \sum_{j=1}^{m} \beta_{i}k(\cdot,\boldsymbol{y}_{\boldsymbol{j}})$ the dot product is defined by

$$<f,g>= \sum_{i=1}^{n} \sum_{j=1}^{m} \alpha_{i}\beta_{j}k(\boldsymbol{x}_{\boldsymbol{i}},\boldsymbol{y}_{\boldsymbol{j}})$$

which satisfies the so called reproducing property $<k\left( \cdot,\boldsymbol{x} \right),f>=f(\boldsymbol{x})$ and therefore $<k\left( \cdot,\boldsymbol{x} \right),k\left( \cdot,\boldsymbol{y} \right)>=k(\boldsymbol{x,y})$. After completion we turn our feature space into a Hilbert space $H$, the reproducing kernel Hilbert space induced by the kernel function $k$[17].

Kernel principal component analysis

Given a feature space $H$ related to the input domain by a kernel feature map

|  | $\phi:\mathcal{X \to}H$ $\boldsymbol{x} \mapsto\phi\left( \boldsymbol{x} \right):=K(\cdot,\boldsymbol{x})$ |  |
| --- | --- | --- |

which is possible non-linear. Let define

|  | $\overline{\phi}:=\frac{1}{n}\sum_{i=1}^{n} \phi\left( \boldsymbol{x}_{\boldsymbol{i}} \right)$ |  |
| --- | --- | --- |
| then the points | $\tilde{\phi}\left( \boldsymbol{x}_{i} \right)=\phi\left( \boldsymbol{x}_{i} \right)-\overline{\phi}$ |  |

are centered. Let $\tilde{K}$ denote the kernel matrix of centered points $\tilde{K}_{ij}= \left\langle\tilde{\phi}\left( \boldsymbol{x}_{i} \right),\tilde{\phi}\left( \boldsymbol{x}_{j} \right) \right\rangle$. We cannot compute $\tilde{K}$explicitly, however, according to Scholkopf and Smola[17] it can be expressed in terms of its noncentered counterpart $K$. Using the vector $\boldsymbol{1}_{n}=\left( 1, \ldots, 1 \right)^{'}$we can get the expression

|  | $\tilde{K}=K-\frac{1}{n}K {\boldsymbol{1}_{n}\boldsymbol{1}}_{n}^{'}-{\frac{1}{n}\boldsymbol{1}_{n}\boldsymbol{1}}_{n}^{'}K+\frac{1}{n^{2}}\left( \boldsymbol{1}_{n}^{'}K \boldsymbol{1}_{n} \right)\boldsymbol{1}_{\boldsymbol{n}}\boldsymbol{1}_{n}^{'}$ |  |
| --- | --- | --- |

In $H$ the covariance matrix takes the form

|  | $\tilde{C}=\frac{1}{n}\sum_{j=1}^{n} \tilde{\phi}\left( \boldsymbol{x}_{j} \right)\tilde{\phi}\left( \boldsymbol{x}_{j} \right)^{'}$ |  |
| --- | --- | --- |

If $H$ is infinite-dimensional, we think of $\tilde{\phi}\left( \boldsymbol{x}_{j} \right)\tilde{\phi}\left( \boldsymbol{x}_{j} \right)^{'}$ as a linear operator on $H$, mapping

|  | $\boldsymbol{x} \mapsto\phi\left( \boldsymbol{x}_{\boldsymbol{j}} \right) \left\langle\phi\left( \boldsymbol{x}_{\boldsymbol{j}} \right),\boldsymbol{x} \right\rangle.$ |  |
| --- | --- | --- |

We have to find eigenvalues $\tilde{\lambda}\geq0$ and nonzero eigenvectors $\tilde{\boldsymbol{V}}\in H\backslash\{0\}$satisfying

|  | $\tilde{\boldsymbol{C}}\tilde{\boldsymbol{V}}\boldsymbol{=}\tilde{\boldsymbol{\lambda}}\tilde{\boldsymbol{V}}$ |  |
| --- | --- | --- |

To find the solution we solve the dual eigenvalue problem

|  | $K\tilde{\boldsymbol{\alpha}}\boldsymbol{=}n\tilde{\lambda}\tilde{\boldsymbol{\alpha}}$ |  |
| --- | --- | --- |

with $\tilde{\boldsymbol{\alpha}}$ being the expansion coefficients of an eigenvector in terms of the centered points

|  | $\tilde{\boldsymbol{V}}= \sum_{i=1}^{n} \tilde{\alpha_{i}} \tilde{\phi}\left( \boldsymbol{x}_{i} \right)$ |  |
| --- | --- | --- |

The solution ${\tilde{\boldsymbol{\alpha}}}^{\boldsymbol{k}}$, $k=1,\ldots,r$, is normalized by normalizing the corresponding vector ${\tilde{\boldsymbol{V}}}^{k}$in $H$,

which translates into

|  | $\boldsymbol{\lambda}_{\boldsymbol{k}}\left\langle{\tilde{\boldsymbol{\alpha}}}^{k}\boldsymbol{,}{\tilde{\boldsymbol{\alpha}}}^{k} \right\rangle=1$ |  |
| --- | --- | --- |

To find the coordinates of a test point $\boldsymbol{s}$, with an image $\phi\left( \boldsymbol{s} \right)$ in $H$, we need to compute projections of the centered image of ***s*** into the subspace in $H$ defined by the eigenvectors ${\tilde{\boldsymbol{V}}}^{k}$, $k=1,\ldots,r$. For this, observe that:

$$\left\langle\phi\left( \boldsymbol{s} \right)-\bar{\phi}, {\tilde{\boldsymbol{V}}}^{k} \right\rangle=\sum_{i=1}^{n} \tilde{\alpha_{i}} \left\langle\phi\left( \boldsymbol{s} \right)-\bar{\phi},\phi\left( \boldsymbol{x}_{\boldsymbol{i}} \right)-\bar{\phi} \right\rangle$$

Introducing the vector

$$\boldsymbol{Z}=\left( K\left( \boldsymbol{s},\boldsymbol{x}_{i} \right) \right)_{n\text{x}1}$$

we have

$$\left( \left\langle\tilde{\phi} \left( \boldsymbol{s} \right), {\tilde{\boldsymbol{V}}}^{k} \right\rangle\right)_{1 \text{x} r}=\left( \boldsymbol{Z}^{'}-{\frac{1}{n}\boldsymbol{1}}_{n}^{'}K \right)\left( \boldsymbol{1}_{n}-{\frac{1}{n}\boldsymbol{1}_{n}\boldsymbol{1}}_{n}^{'} \right)\tilde{\boldsymbol{V}}$$

where $\tilde{\boldsymbol{V}}$ is a $n \text{x} r$ matrix whose columns are the eigenvectors ${\tilde{\boldsymbol{V}}}^{1}, \ldots, {\tilde{\boldsymbol{V}}}^{r}$.
