# Supplementary figures and images for: SVM-RFE: selection and visualization of the most relevant features through non-linear kernels
Source: BMC Bioinformatics. 2018 Nov 19;19:432. doi: 10.1186/s12859-018-2451-4 (PMC6245920; doi:10.1186/s12859-018-2451-4)

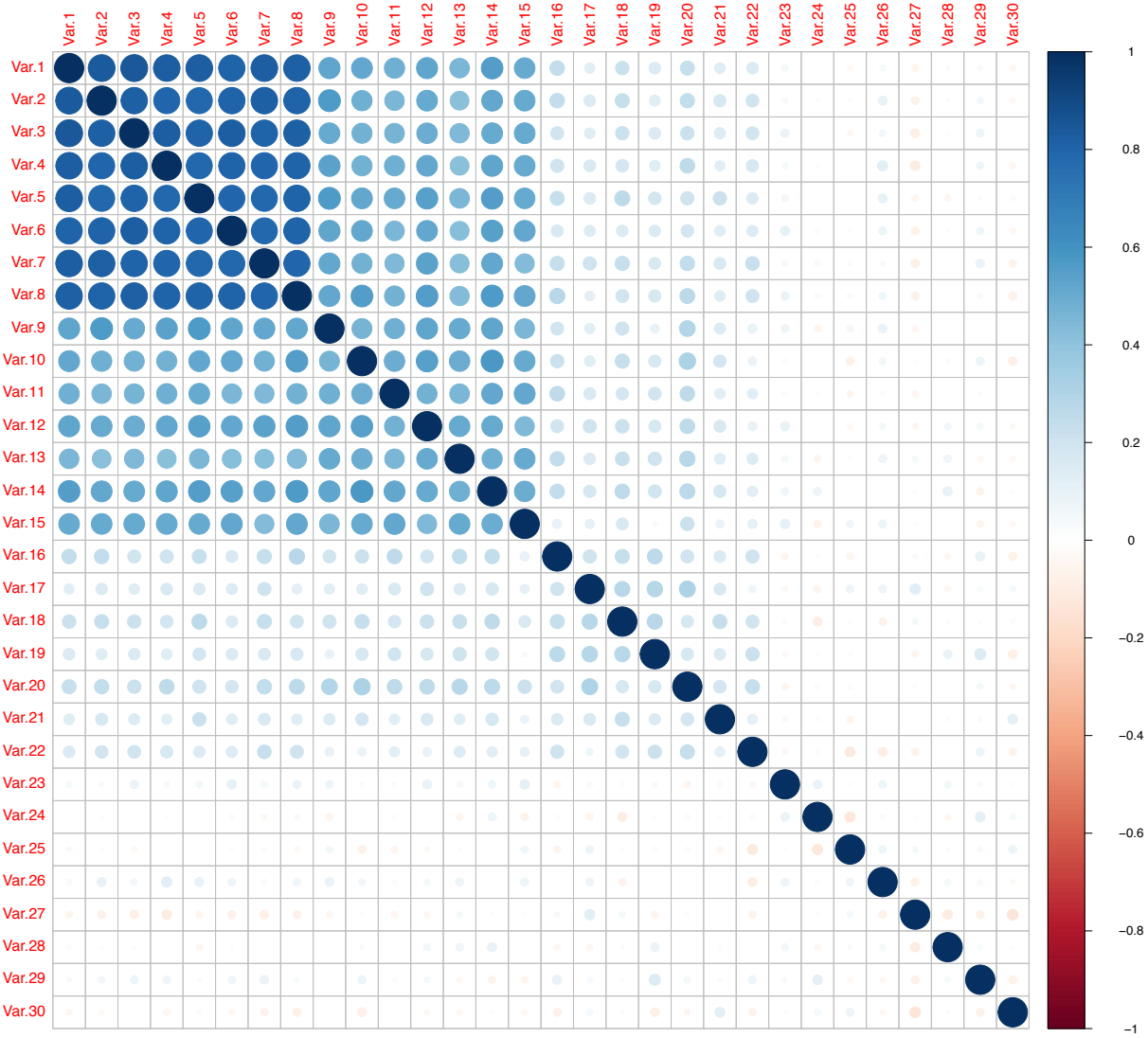

Supplement: Supplementary file 2 — Pearson correlation matrix of the 30 variables simulated. (PDF 131 kb) [file 12859_2018_2451_MOESM2_ESM.pdf]

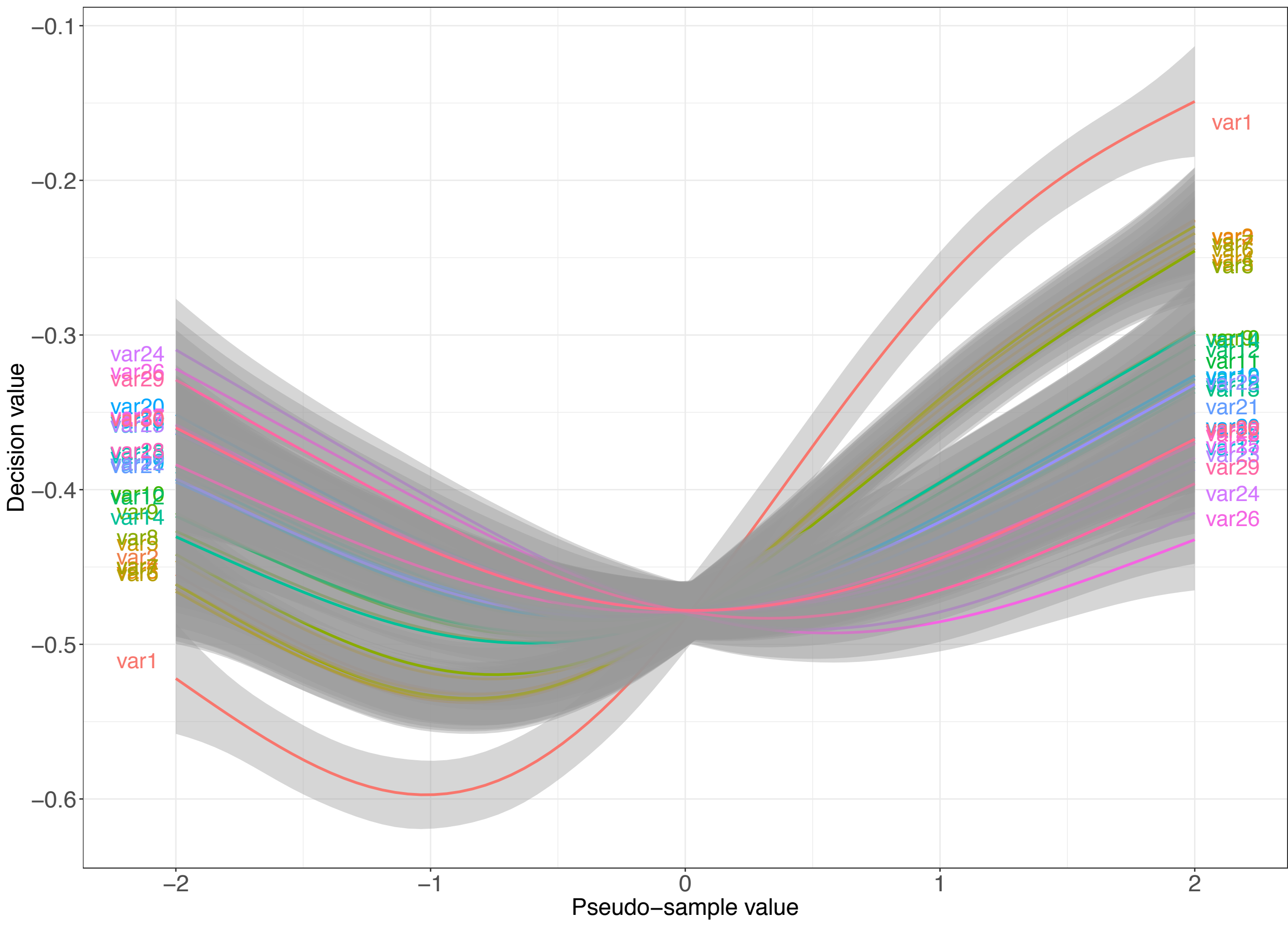

Supplement: Supplementary file 4 — Visualization of RFE-pseudo-samples results for Scenario 1. Scenario 1 (being Variable 1 the relevant variable) results for all 100 simulated datasets, all 30 variables and first iteration of the RFE-pseudo-samples algorithm. The pseudo-samples distribution for each variable is shown with a non-parametric local regression estimation (LOESS) with the corresponding 95% confidence interval. (PDF 61 kb) [file 12859_2018_2451_MOESM4_ESM.pdf]

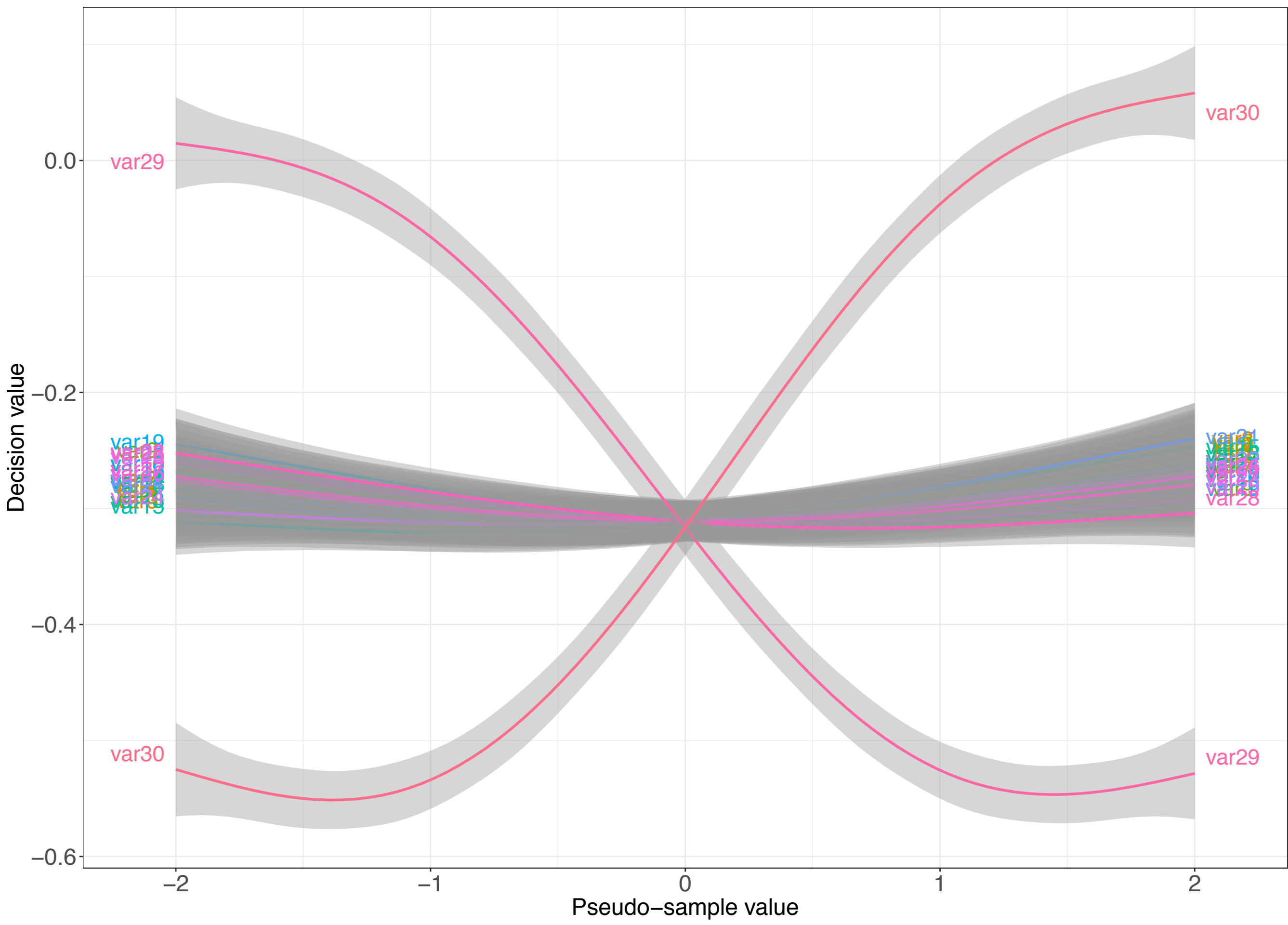

Supplement: Supplementary file 5 — Visualization of RFE-pseudo-samples results for Scenario 2. Scenario 2 (being Variable 29 and 30 the relevant variables) results for all 100 simulated datasets, all 30 variables and first iteration of the RFE-pseudo-samples algorithm. The pseudo-samples distribution for each variable is shown with a non-parametric local regression estimation (LOESS) with the corresponding 95% confidence interval. (PDF 59 kb) [file 12859_2018_2451_MOESM5_ESM.pdf]

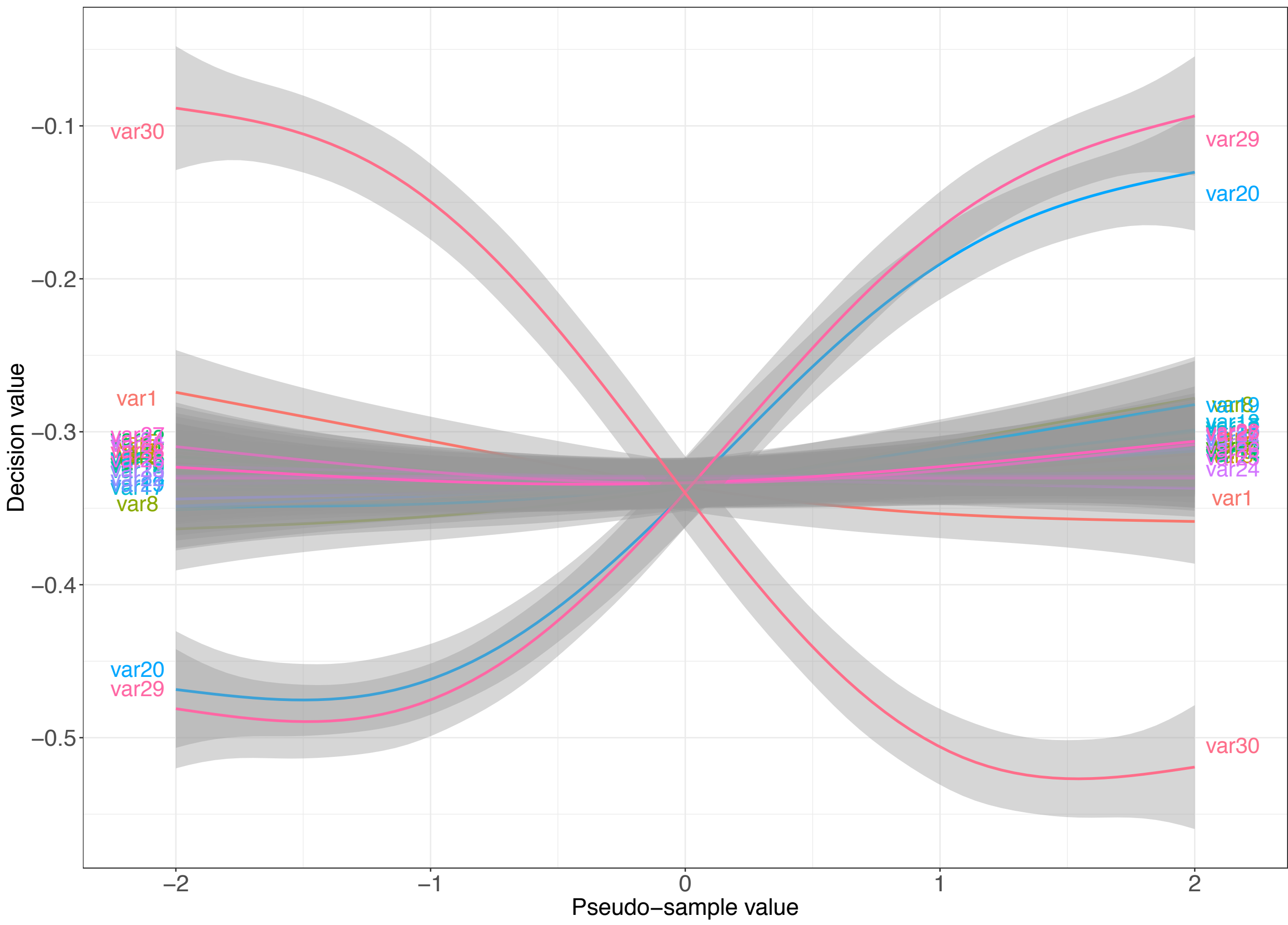

Supplement: Supplementary file 6 — Visualization of RFE-pseudo-samples results for Scenario 3. Scenario 3 (being Variable 1, 8, 20, 29 and 30 the relevant variables) results for all 100 simulated datasets, all 30 variables and first iteration of the RFE-pseudo-samples algorithm. The pseudo-samples distribution for each variable is shown with a non-parametric local regression estimation (LOESS) with the corresponding 95% confidence interval. (PDF 57 kb) [file 12859_2018_2451_MOESM6_ESM.pdf]

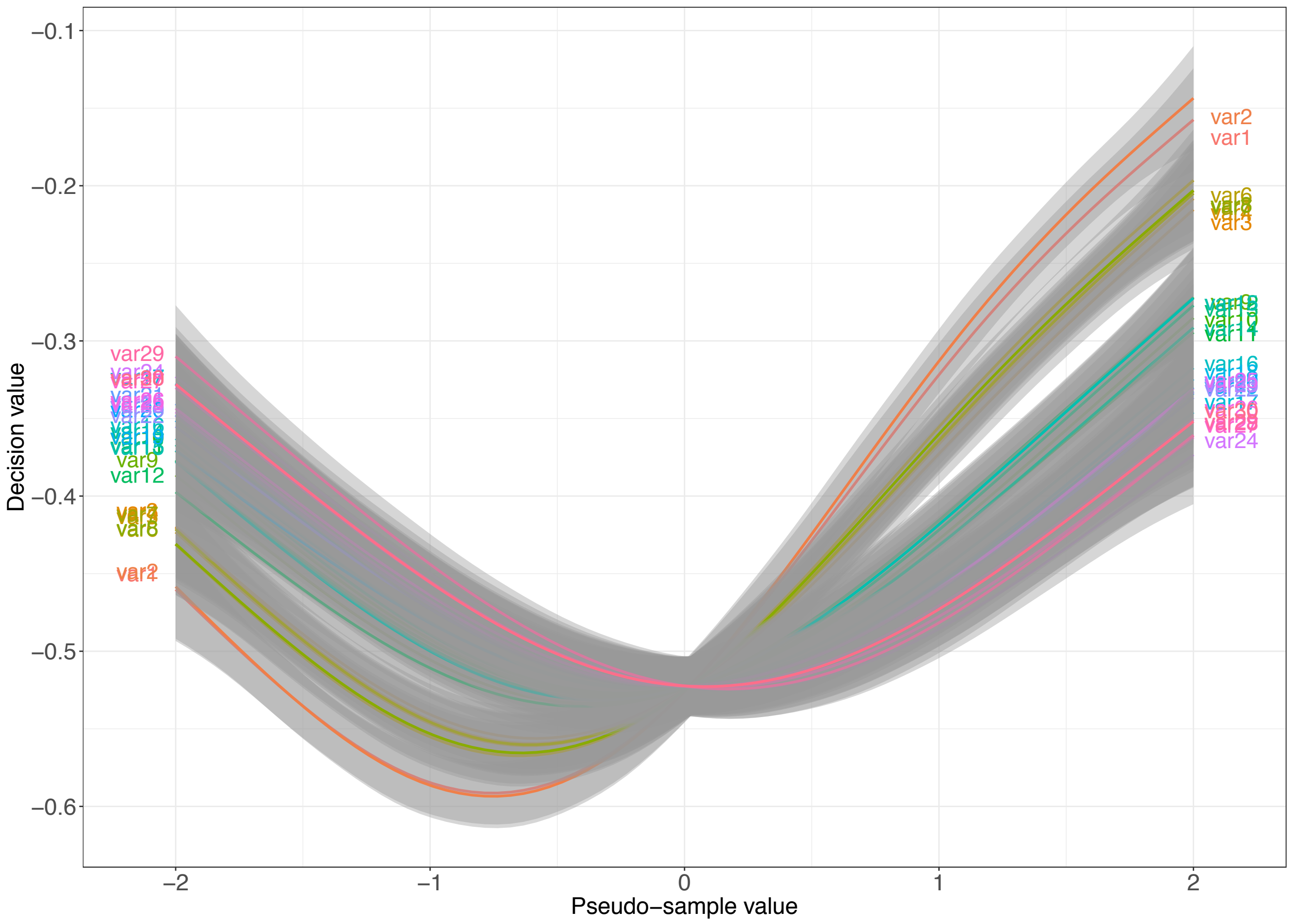

Supplement: Supplementary file 7 — Visualization of RFE-pseudo-samples results for Scenario 4. Scenario 4 (being Variable 1 and 2 the relevant variables) results for all 100 simulated datasets, all 30 variables and first iteration of the RFE-pseudo-samples algorithm. The pseudo-samples distribution for each variable is shown with a non-parametric local regression estimation (LOESS) with the corresponding 95% confidence interval. (PDF 61 kb) [file 12859_2018_2451_MOESM7_ESM.pdf]

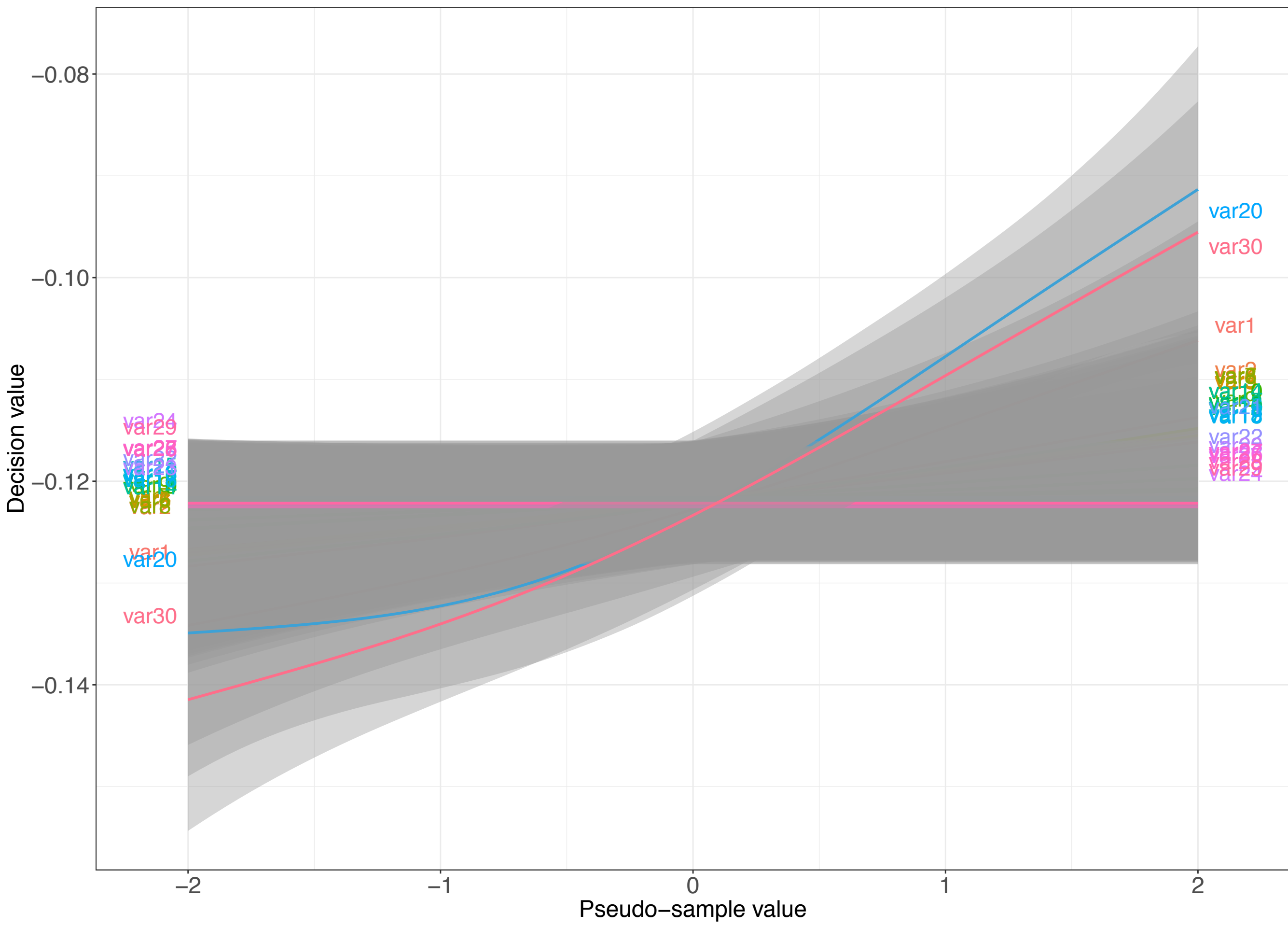

Supplement: Supplementary file 8 — Visualization of RFE-pseudo-samples results for Scenario 5. Scenario 5 (being Variable 1, 20 and 30 the relevant variables) results for all 100 simulated datasets, all 30 variables and first iteration of the RFE-pseudo-samples algorithm. The pseudo-samples distribution for each variable is shown with a non-parametric local regression estimation (LOESS) with the corresponding 95% confidence interval. (PDF 53 kb) [file 12859_2018_2451_MOESM8_ESM.pdf]

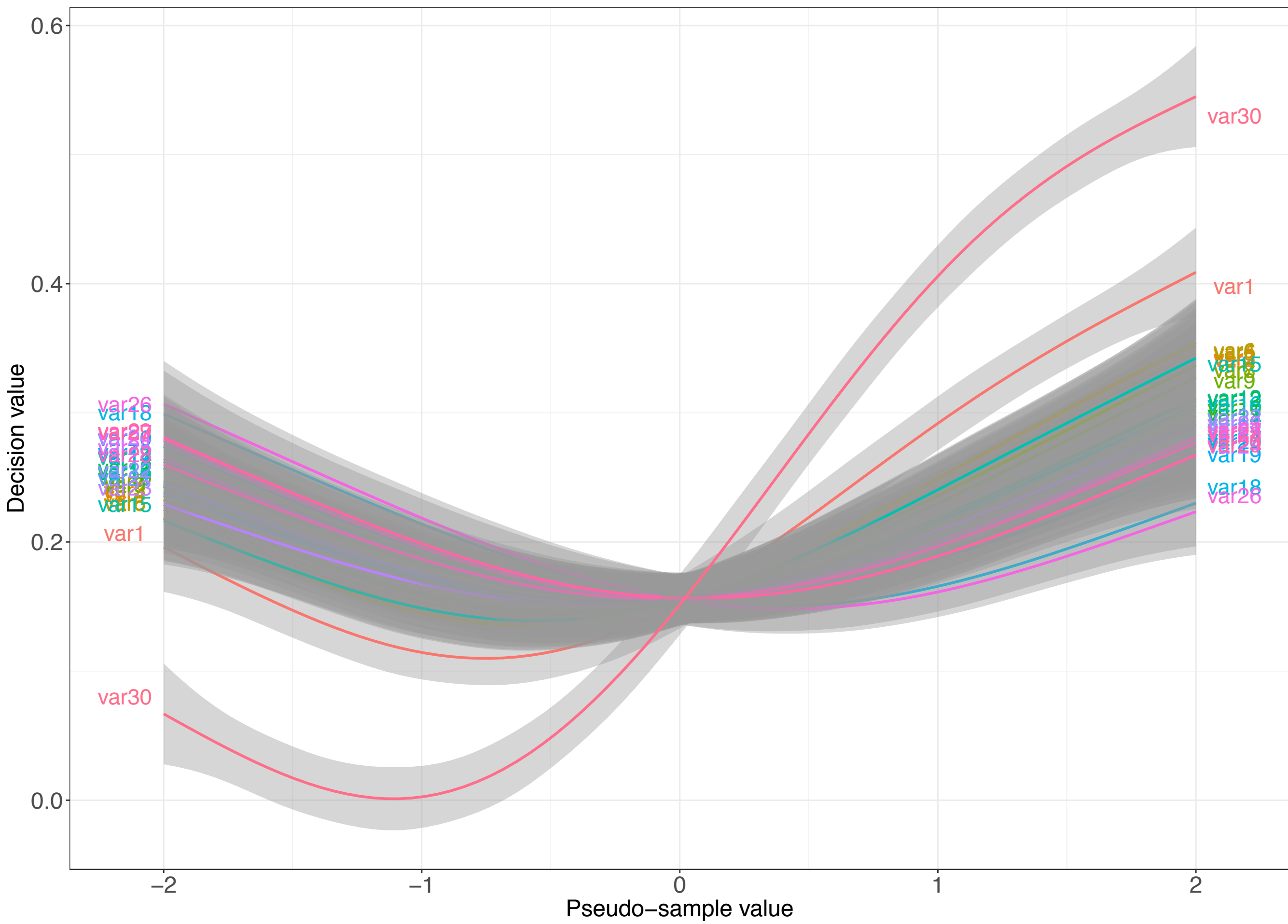

Supplement: Supplementary file 9 — Visualization of RFE-pseudo-samples results for Scenario 6. Scenario 6 (being Variable 1 and 30 the relevant variables) results for all 100 simulated datasets, all 30 variables and first iteration of the RFE-pseudo-samples algorithm. The pseudo-samples distribution for each variable is shown with a non-parametric local regression estimation (LOESS) with the corresponding 95% confidence interval. (PDF 60 kb) [file 12859_2018_2451_MOESM9_ESM.pdf]

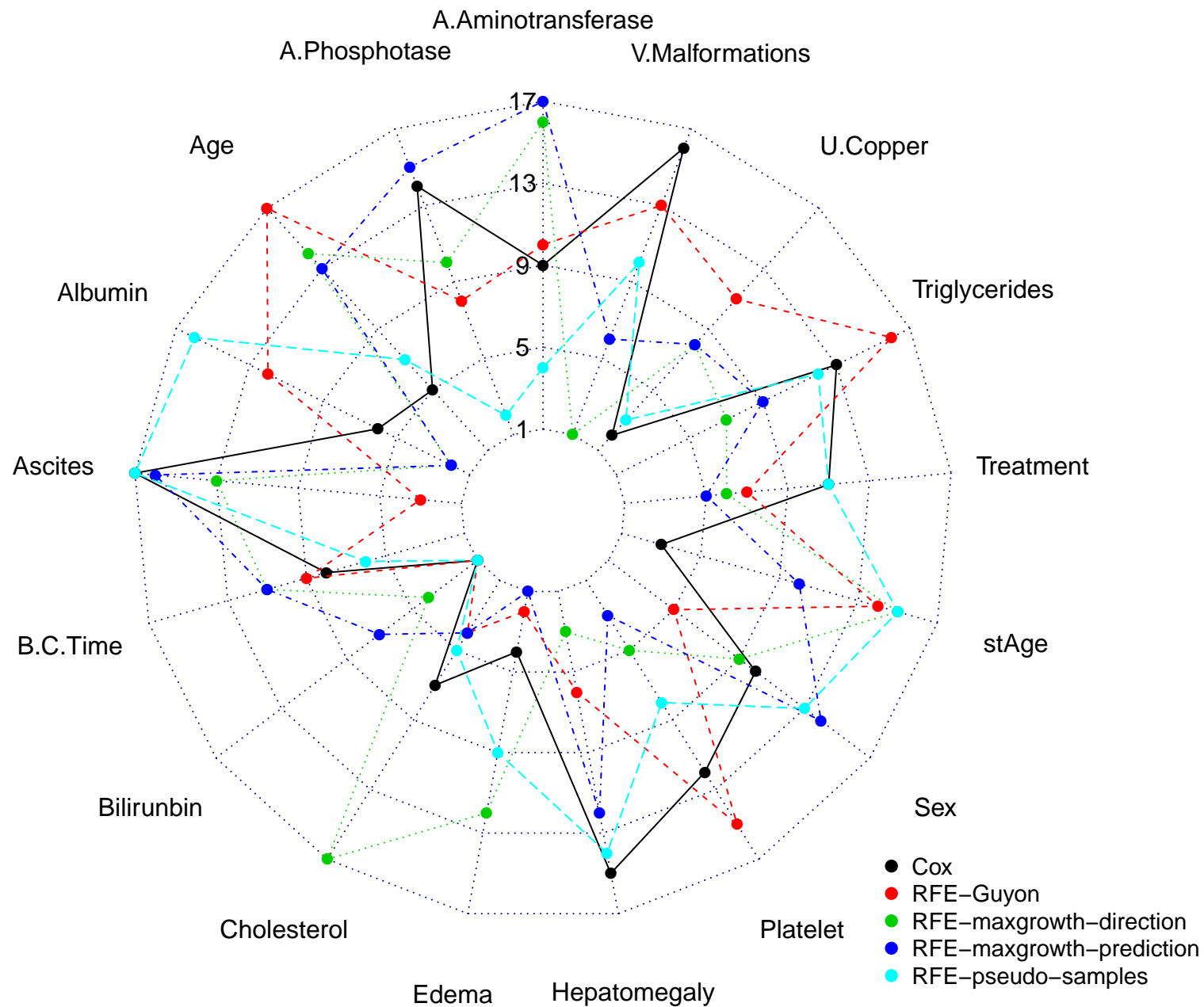

Supplement: Supplementary file 10 — Results for PBC dataset comparing the four RFE algorithms and the Cox model. (PDF 6 kb) [file 12859_2018_2451_MOESM10_ESM.pdf]

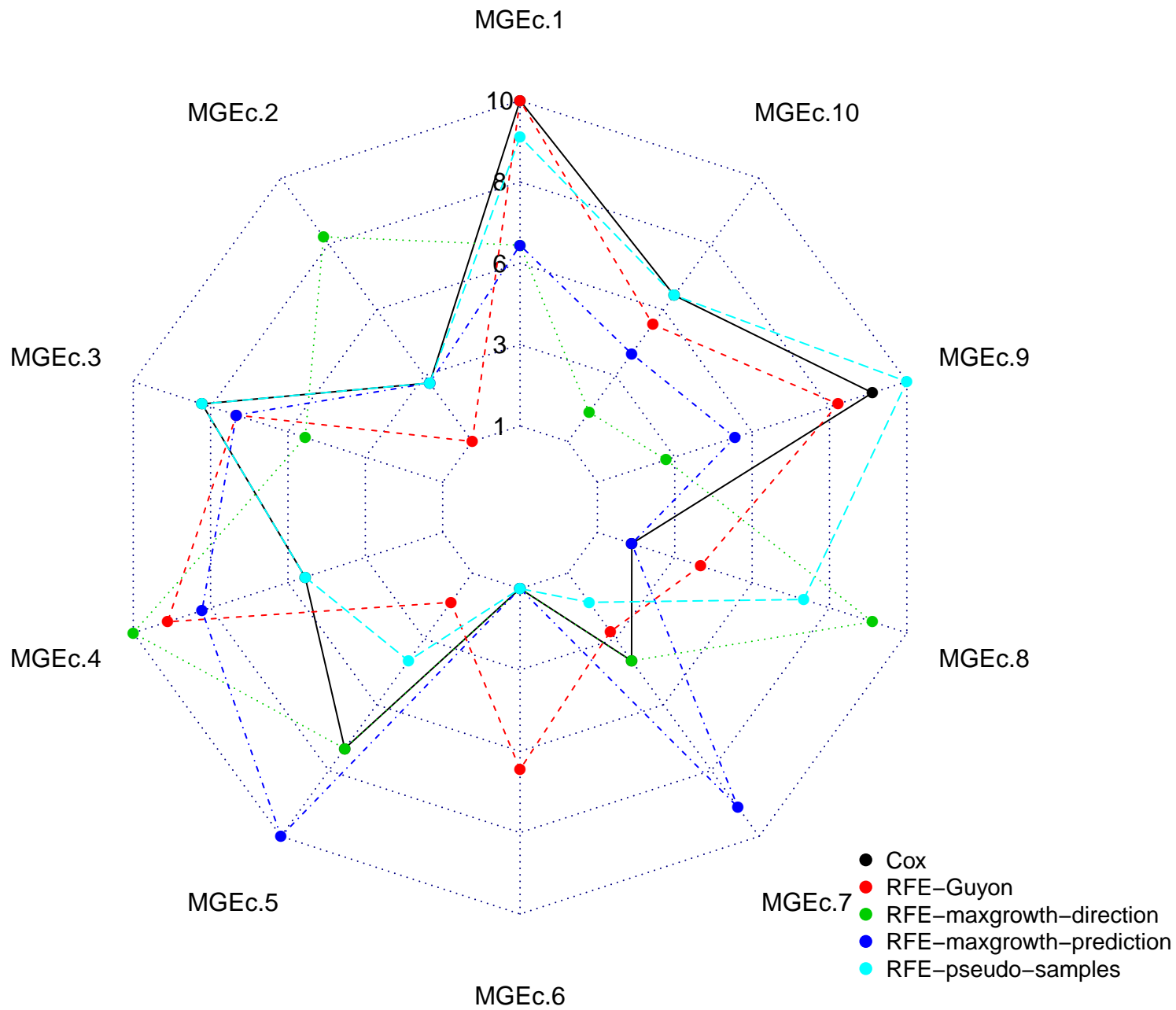

Supplement: Supplementary file 11 — Results for DLBCL dataset comparing the four RFE algorithms and the Cox model. (PDF 5 kb) [file 12859_2018_2451_MOESM11_ESM.pdf]

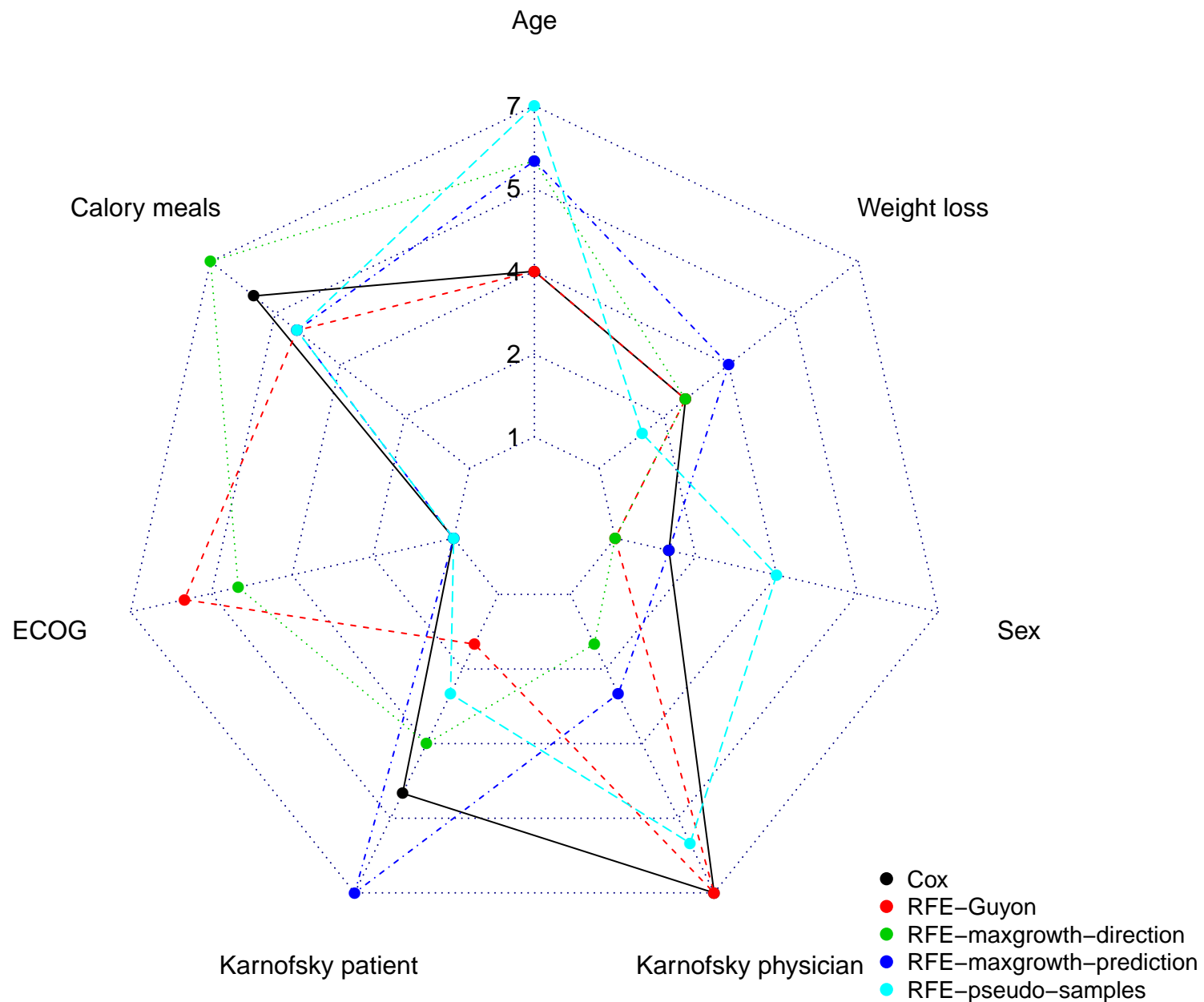

Supplement: Supplementary file 12 — Results for Lung dataset comparing the four RFE algorithms and the Cox model. (PDF 5 kb) [file 12859_2018_2451_MOESM12_ESM.pdf]
