# Supplementary material for: SVM-RFE: selection and visualization of the most relevant features through non-linear kernels
Source: BMC Bioinformatics. 2018 Nov 19;19:432. doi: 10.1186/s12859-018-2451-4 (PMC6245920; doi:10.1186/s12859-018-2451-4)
Supplement: Supplementary file 3 — Probabilistic support vector machine methodology. (DOCX 36 kb) [file 12859_2018_2451_MOESM3_ESM.docx]

Probabilistic SVM

The pSVM accounts for class membership through a hinge loss and estimates probabilities using the $\epsilon$-insensitive cost function. Given an observation $i$ we define the pair ${(\boldsymbol{x}}_{i},l_{i})$ as the learning dataset of input vectors along with their corresponding classes group. The classes can be defined as

|  | $l_{i}=y_{i}\in\left\{ \pm1 \right\}\text{ for} i=1, \ldots.,n$ $l_{i}=p_{i}\in\left[ 0,1 \right]\text{ for} i=n+1, \ldots.,m$ |  |
| --- | --- | --- |

where $n$ is the number of observations with known classes (perfectly definite), $(m-n-1)$the number of observations with uncertain classes and $p_{i}$ is the associated uncertainty about $\boldsymbol{x}_{i}$ in a regression setting. The posterior probability for class 1 is given by

|  | $p_{i}=Prob\left( Y_{i}=1 \right\vert\boldsymbol{X}_{i}=\boldsymbol{x}_{i})$ |  |
| --- | --- | --- |

The associated optimization problem is

|  | $\underset{\boldsymbol{w},b}{\mathrm{minimize}} \frac{1}{2}\left\Vert\boldsymbol{w} \right\Vert^{2}$ $\text{subject to } y_{i}\left( \left\langle\boldsymbol{w}, \boldsymbol{x}_{i} \right\rangle+b \right)\geq1, i= 1, \ldots, n$ $z_{i}^{-} \leq\left\langle\boldsymbol{w}, \boldsymbol{x}_{i} \right\rangle+b \leq z_{i}^{+}, i= n+1, \ldots, m$ |  |
| --- | --- | --- |

where $z_{i}^{-}$ and $z_{i}^{+}$ are boundaries depending on $p_{i}$. If $n=m$ the problem is exactly the same to hard margin SVM. Allowing to misclassification in classes, slack variables $\xi_{i}$ are introduced. Then the optimization problem can be rewritten as

|  | $\underset{\boldsymbol{w},\boldsymbol{\xi},\boldsymbol{\xi}^{-},\boldsymbol{\xi}^{+},b}{\mathrm{minimize}} \frac{1}{2}\left\Vert\boldsymbol{w} \right\Vert^{2} +C \sum_{i=1}^{n} \xi_{i}+\tilde{C} \sum_{i=n+1}^{m} \left( \xi_{i}^{-}+ \xi_{i}^{+} \right)$ $\text{subject to} y_{i}\left( \left\langle\boldsymbol{w}, \boldsymbol{x}_{\boldsymbol{i}} \right\rangle+b \right)\geq1-\xi_{i}, i= 1, \ldots, n$ $z_{i}^{-} -\xi_{i}^{-}\leq\left\langle\boldsymbol{w}, \boldsymbol{x}_{i} \right\rangle+b \leq z_{i}^{+}+\xi_{i}^{+}, i= n+1, \ldots, m$ $\xi_{i}\geq0, i=1,\ldots,n$ $\xi_{i}^{-}\geq0, i=n+1,\ldots,m$ $\xi_{i}^{+}\geq0, i=n+1,\ldots,m$ |  |
| --- | --- | --- |

For the censored data the probability can be computed based on the remaining follow-up time, i.e., based on the proportional follow-up time[23], computing the probability $p_{i}$ for censored data and subsequently $z_{i}^{-}$and $z_{i}^{+}$ as $\frac{T_{i}}{\tau}$, being $\tau$ the maximum follow-up time established in the study cohort and $T_{i}$ the observed follow-up time. For the events and the non-events at the end of the follow-up period this value is fixed to 1.
